# Prokineticin-2 upregulation during neuronal injury mediates a compensatory protective response against dopaminergic neuronal degeneration

**DOI:** 10.1038/ncomms12932

**Published:** 2016-10-05

**Authors:** Richard Gordon, Matthew L. Neal, Jie Luo, Monica R. Langley, Dilshan S. Harischandra, Nikhil Panicker, Adhithiya Charli, Huajun Jin, Vellareddy Anantharam, Trent M. Woodruff, Qun-Yong Zhou, Anumantha G. Kanthasamy, Arthi Kanthasamy

**Affiliations:** 1 Parkinson Disorders Research Program, Iowa Center for Advanced Neurotoxicology, Department of Biomedical Sciences, Iowa State University, Ames, Iowa 50011, USA; 2 School of Biomedical Sciences, The University of Queensland, Brisbane, Queensland 4072, Australia; 3 Department of Pharmacology, 363D Med Surge 2, University of California, Irvine, California 92697, USA

## Abstract

Prokineticin-2 (PK2), a recently discovered secreted protein, regulates important physiological functions including olfactory biogenesis and circadian rhythms in the CNS. Interestingly, although PK2 expression is low in the nigral system, its receptors are constitutively expressed on nigrostriatal neurons. Herein, we demonstrate that PK2 expression is highly induced in nigral dopaminergic neurons during early stages of degeneration in multiple models of Parkinson's disease (PD), including PK2 reporter mice and MitoPark mice. Functional studies demonstrate that PK2 promotes mitochondrial biogenesis and activates ERK and Akt survival signalling pathways, thereby driving neuroprotection. Importantly, PK2 overexpression is protective whereas PK2 receptor antagonism exacerbates dopaminergic degeneration in experimental PD. Furthermore, PK2 expression increased in surviving nigral dopaminergic neurons from PD brains, indicating that PK2 upregulation is clinically relevant to human PD. Collectively, our results identify a paradigm for compensatory neuroprotective PK2 signalling in nigral dopaminergic neurons that could have important therapeutic implications for PD.

## Abstract (web summary)

Prokineticin-2 (PK2) is a secreted protein involved in a number of physiological functions. Here, the authors find that PK2 expression increases in surviving DA neurons from Parkinson's disease patients, and show it protects against dopaminergic degeneration in PD mouse models.

## Graphical abstract

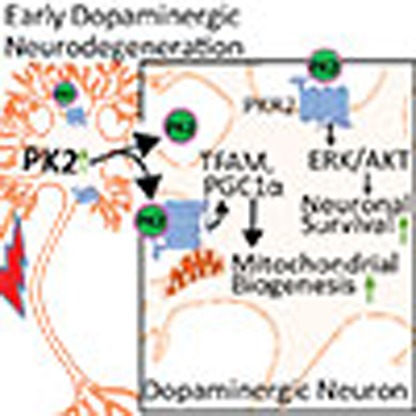

Prokineticin-2 (PK2), of the AVIT protein family, regulates diverse physiological processes including hematopoiesis, angiogenesis, reproductive functions and pain perception^1^,^2^,^3^,^4^,^5^,^6^. In the brain, PK2 regulates circadian rhythms by functioning as an output molecule from the suprachiasmatic nucleus (SCN) and neurogenesis by directing the migration of progenitor cells from the subventricular zone during olfactory bulb biogenesis^1^,^5^,^7^. Recent evidence also links PK2 signalling to thermoregulation and energy expenditure via the hypothalamus^8^. Constitutive expression of PK2 is largely restricted to these aforementioned regions, however, its two highly similar G-protein-coupled receptors (GPCR), PKR1 and PKR2, are ubiquitously and differentially expressed across the central nervous system (CNS), reflecting PK2's distinct functions throughout the brain^9^.

While investigating changes in gene expression during dopaminergic cell death via PCR array, we surprisingly found that PK2 mRNA was highly induced during dopaminergic (DAergic) cell death triggered by tumour necrosis factor alpha (TNFα)^10^. We therefore hypothesized that PK2 is a novel signalling mediator secreted during dopaminergic degeneration. Supporting this notion, our mouse models of PD revealed that PK2 is induced in nigral dopaminergic neurons early during degeneration, before the onset of motor deficits. We also found that PK2 expression is elevated in the substantia nigra (SN) of PD patients, corroborating PK2's clinical relevance in human PD. PK2 activates PKR1 and PKR2, both of which are differentially expressed in the CNS^4^,^11^,^12^,^13^. We found that PKR2 is constitutively expressed in dopaminergic neurons of the nigrostriatal system in mice and human PD cases, suggesting that PK2 signalling via PKR2 could be relevant during dopaminergic degeneration. Indeed, our functional studies using PK2 overexpression and recombinant PK2 (rPK2) demonstrated that PK2 signalling protects against oxidative stress, mitochondrial dysfunction and dopaminergic degeneration induced by the Parkinsonian neurotoxicant MPP^+^. Blocking PK2 signalling using a PK2 receptor antagonist augmented dopaminergic degeneration in a PD mouse model, while overexpression of PK2 by AAV gene delivery was neuroprotective. Given that prokineticin GPCRs are druggable targets and that PK2 signalling is neuroprotective against dopaminergic degeneration, our results have potential therapeutic implications for mitigating dopaminergic neuron loss in PD using prokineticin receptor agonists.

## Results

### PK2 is induced during MPP^+^- and TNFα-induced N27 cell death

**Figure 1:**
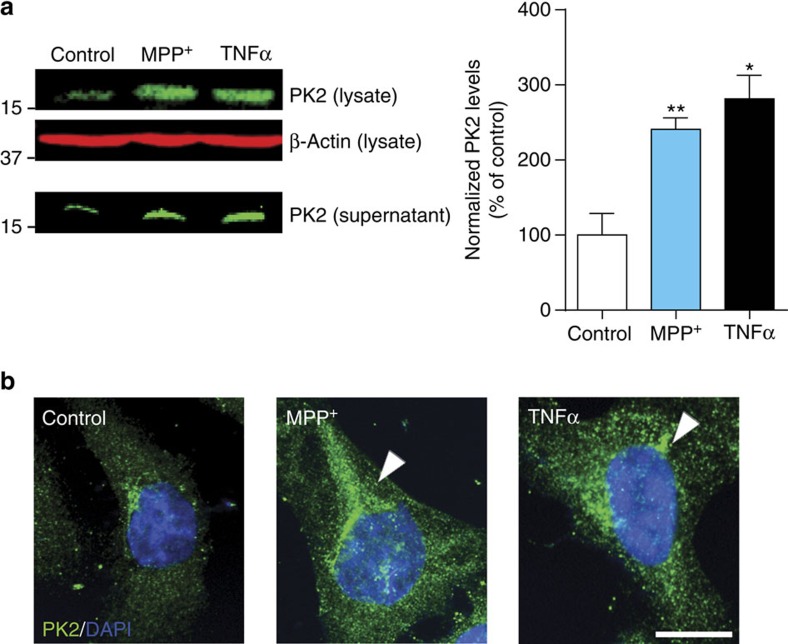
Prokineticin-2 is upregulated and secreted following neurotoxic stress in N27 dopaminergic neuronal cells. (**a**) Upregulation and release of PK2 in dopaminergic N27 cells. Representative (left) western blot analysis of intracellular and secreted PK2 protein following treatment of N27 cells with TNFα (30 ng ml^−1^) and MPP^+^ (300 μM) for 12 h. At the end of treatment, supernatants were collected and concentrated using a Vivaspin centrifugal concentrator with a 3-kDa molecular weight cutoff. Experiments were repeated twice with *n*=3. Data represented as mean±s.e.m. and asterisks denote a significant (*P<0.05 and ^**^P<0.01) difference between control and TNFα- or MPP^+^-treated groups using one-way ANOVA with Bonferroni post-test comparison. (**b**) Localization of PK2 (green) in N27 cells was determined via IHC after exposure to TNFα and MPP^+^ for 12 h. Hoechst dye stained the nuclei (blue). Cytosolic expression of PK2 was localized to the peri-nuclear region (white arrows). Scale bar, 10 μm.

Using qPCR array, we serendipitously discovered that PK2 mRNA expression is highly induced (∼7-fold over control) early during dopaminergic cell death (Supplementary Table. 1). Thus, we hypothesized that PK2 is likely a compensatory bioactive signalling mediator secreted early during dopaminergic degeneration. To validate this hypothesis, we determined both TNFα (30 ng ml^−1^) and the Parkinsonian toxicant MPP^+^ (300 μM) increased PK2 protein expression in N27 cells (Fig. 1a). PK2 supernatant levels also increased, demonstrating that PK2 is both upregulated and secreted by dopaminergic neuronal cells following neurotoxic stress. A rabbit polyclonal antibody that recognizes an epitope within the internal region of PK2 was used for immunofluorescence detection of PK2 expression and localization in N27 dopaminergic cells (Fig. 1b). Typical of a secreted protein, PK2 accumulated prominently around the perinuclear zone (arrow heads). Indeed, PK2 contains a signal peptide at its N-terminus targeting it for constitutive secretion^12^,^14^. Our results identify PK2 as an inducible secreted mediator during early stages of neurotoxic stress.

### PK2 is induced in nigral DAergic neurons in experimental PD

**Figure 2:**
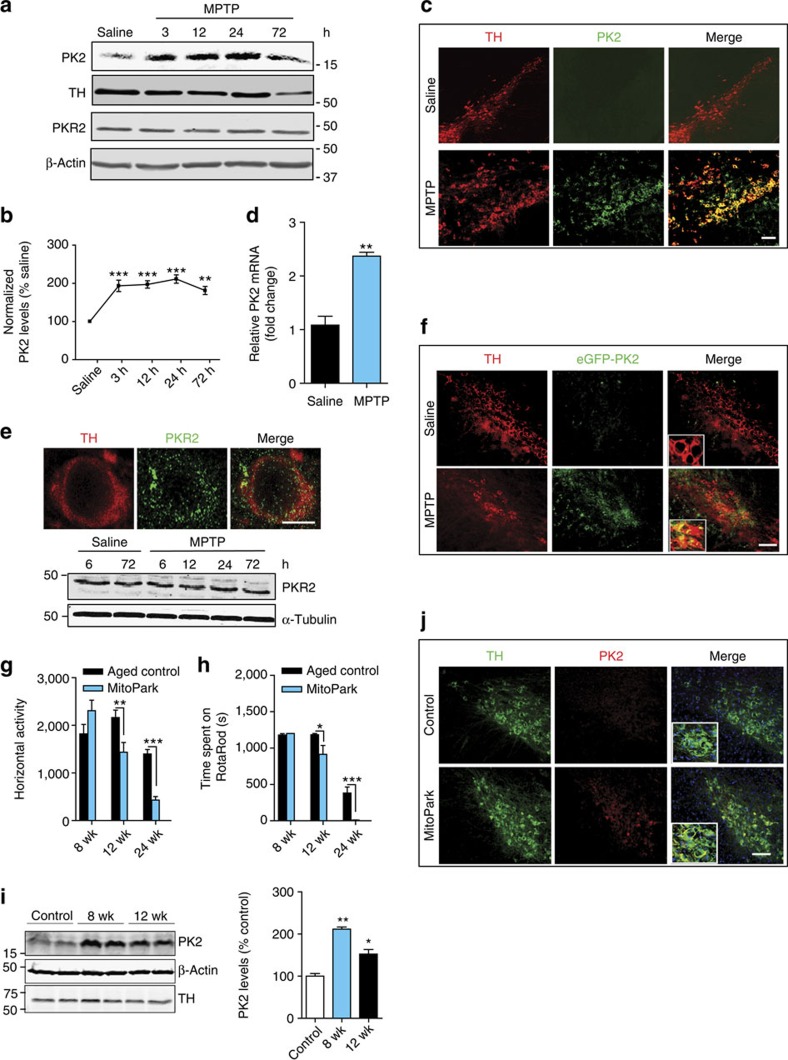
Prokineticin-2 is highly induced in nigral dopaminergic neurons in animal models of Parkinson's disease. (**a**–**d**) Mice were injected with four MPTP doses (18 mg kg^−1^, i.p.) at 2-h intervals. (**a**,**b**) Representative western blots (**a**) of PK2, PKR2 and TH expression in nigral lysates. Mice were euthanized at 3, 12, 24 and 72 h post MPTP. Experiments were repeated three times with three mice per group and quantified by densitometric analysis after normalization to β-Actin (**b**). (**c**) Double-labelling IHC for PK2 and TH in mouse nigral sections stained for TH (red) and PK2 (green). Strong PK2 expression occurred in TH^+^ neurons along the nigral tract. Scale bar, 40 μm. (**d**) Real-time PCR for PK2 mRNA expression in the SN, 24 h post-MPTP treatment, with 18S as the internal standard for normalization. Data represented by group mean±s.e.m. from experiments repeated twice with *n*=4. Asterisks denote a significant difference between control and MPTP treatment (^**^P<0.01) using Student's *t*-test. (**e**) Top: double-labelling IHC shows expression of PKR2 (green) on TH^+^ neurons. Image magnification, × 60. Scale bar, 10 μm. Bottom: western blot for PKR2 in mouse nigral lysates. Mice receiving four doses of MPTP (18 mg kg^−1^, i.p.) were euthanized at the indicated time points. α-Tubulin was used as the loading control. Representative blots indicate that PKR2 is constitutively expressed. (**f**) Visualization of PK2 using GENSAT Prok2-eGFP reporter transgenic mice. Coronal sections from saline- or MPTP-treated transgenic mice were stained for TH (red) with eGFP directly imaged at 488 nm excitation. Image magnification, × 20. Scale bar, 40 μm. Representative images are shown and experiments were repeated at least three times. (**g**–**j**) PK2 induced in nigral dopaminergic neurons in transgenic MitoPark mice. Horizontal activity (**g**) and rotarod performance (**h**) showing no significant differences in motor function at 8 weeks and onset of motor deficits at 12 weeks (*n*=7–10 mice per group). Data represented as mean±s.e.m. (*P<0.05, ^**^P<0.01, ^***^P<0.001) using two-way ANOVA. (**i**) Western blot of PK2 and TH expression revealed significantly elevated PK2 in 8- and 12-week MitoPark mice. Quantification of PK2 band is shown on the right. (**j**) IHC staining for TH (green) and PK2 (red) showing increased PK2 expression in nigral dopaminergic neurons at 12 weeks. Scale bar, 40 μm. Data represented as mean±s.e.m. (*P<0.05, ^**^P<0.01, ^***^P<0.001) with an *n*=2–8 using one-way ANOVA with Bonferroni post-test comparison.

Previous studies characterizing PK2 expression in the adult mouse brain reported it either absent or sparse in the ventral midbrain, including the SN^9^,^15^. Since PK2 receptors were constitutively expressed mostly on neurons, it is possible that PK2 ligand expression could mediate specific signalling functions via its cognate receptors. Initially, using the standard acute (4 × 18 mg kg^−1^, 2 h intervals) MPTP model of PD, we verified, in concordance with our *in vitro* results, that MPTP significantly increased PK2 levels in nigral tissue lysates. Western blot (Fig. 2a) and densitometric analysis (Fig. 2b) revealed that upregulation occurred as early as 3 h after MPTP treatment and peaked at 24 h in MPTP-treated mice. Reduced nigral levels of tyrosine hydroxylase (TH), a marker of dopaminergic neurons, was not observed until 3 days post treatment, suggesting MPTP-induced PK2 upregulation precedes dopaminergic degeneration (Fig. 2a). No significant difference in PKR2 expression existed between MPTP-treated and saline-injected control mice (Fig. 2a). We confirmed that PK2 staining co-localized largely with TH-positive (TH^+^) dopaminergic neurons (Fig. 2c). PK2 upregulation was not observed in the striatum or cortex (Supplementary Fig. 1). In saline-treated mice, we observed little or no PK2 expression, and no distinct cellular staining patterns, which supports Cheng *et al*.^16^, who characterized PK2 expression throughout the entire adult mouse brain by *in situ* hybridization without finding significant expression in the SN pars compacta (SNpc). We also confirmed PK2 mRNA upregulation in the nigra (Fig. 2d). Two GPCRs for PK2, prokineticin receptors 1 and 2 (PKR1/2), have been identified and cloned by independent groups^4^,^11^,^17^. We determined their expression in the SN of both saline- and MPTP-treated mice. Cellular PKR2 was highly expressed on dopaminergic neurons and co-localized with TH staining revealing a punctate pattern of PKR2 staining evident at higher magnification (Fig. 2e). Unlike the PK2 ligand, MPTP did not alter the constitutive expression of PKR2 in the SN (Fig. 2e).

To further confirm PK2 upregulation in dopaminergic nigral neurons, we used GENSAT BAC PK2 eGFP transgenic mice [Tg (Prok2-eGFP) FH64Gsat]. This transgenic mouse contains an eGFP reporter transgene and a polyadenylation sequence introduced between the PK2 promoter and the first coding exon of the PK2 gene^18^,^19^. Transcription of the eGFP reporter is driven by PK2 promoter activation, and is terminated at the polyadenylation site, so that reporter expression occurs without any changes in native PK2 expression. Consistent with reported constitutive PK2 expression in the SCN and olfactory bulb^1^,^7^, we observed high eGFP fluorescence in these regions in the adult PK2-eGFP mouse brain (Supplementary Fig. 2A), confirming the utility of this model for our studies. PK2-eGFP mice were treated with MPTP (4 × 20 mg kg^−1^, 2 h intervals). In nigral sections from saline-treated mice, eGFP fluorescence was undetectable (Fig. 2f). However, in MPTP-treated mice at 24 h, strong eGFP fluorescence was evident along the nigral tract with most of the eGFP-positive cells being TH-positive dopaminergic neurons. Some TH-negative cells in the nigral tract were also labelled with eGFP (Fig. 2f), representing less than 5% of the PK2-positive cells in the SN; however, the identity of these PK2-positive cells is unclear. We also confirmed our IHC findings by western blotting PK2 and GFP (Supplementary Fig. 2B). Together, these results demonstrate that PK2 is highly expressed in nigral dopaminergic neurons of PK2-eGFP mice following MPTP-induced degeneration.

The acute MPTP model exhibits rapid dopaminergic degeneration. To verify if PK2 is upregulated in a more gradual and progressive model of dopaminergic degeneration, we utilized the MitoPark mouse, which is an L-DOPA-responsive transgenic model of PD generated by targeted inactivation of the mitochondrial transcription factor A (*Tfam*) gene in dopaminergic neurons. Thus, mitochondrial dysfunction drives progressive dopaminergic degeneration in MitoPark mice over a period of 32 weeks and is accompanied by behavioural motor deficits^20^,^21^. We investigated whether PK2 upregulation occurred at time points preceding the onset of motor deficits in MitoPark mice, which have been reported to begin at around age 12 weeks^20^,^21^. First, we confirmed the time course of MitoPark motor deficits and found no significant motor impairments at 8 weeks compared with age-matched wild-type C57 controls (Fig. 2g,h, Supplementary Fig. 3). PK2 expression increased significantly at 8 and 12 weeks (Fig. 2i). Consistent with our observation that PK2 upregulation precedes dopaminergic neurodegeneration, no significant loss of nigral TH protein was evident in MitoPark mice at 8 weeks (Fig. 2i). We also confirmed that PK2 was localized to TH^+^ nigral dopaminergic neurons in 12-week-old MitoPark mice (Fig. 2j). Overall, these results demonstrate that PK2 is upregulated specifically in nigral dopaminergic neurons following both acute and progressive dopaminergic degeneration. Constitutive PK2 receptor expression on adjacent nigral dopaminergic neurons suggests that PK2 has autocrine/paracrine signalling roles as a compensatory response following dopaminergic neuron injury and degeneration.

### PK2 is elevated in the substantia nigra of PD brains

**Figure 3:**
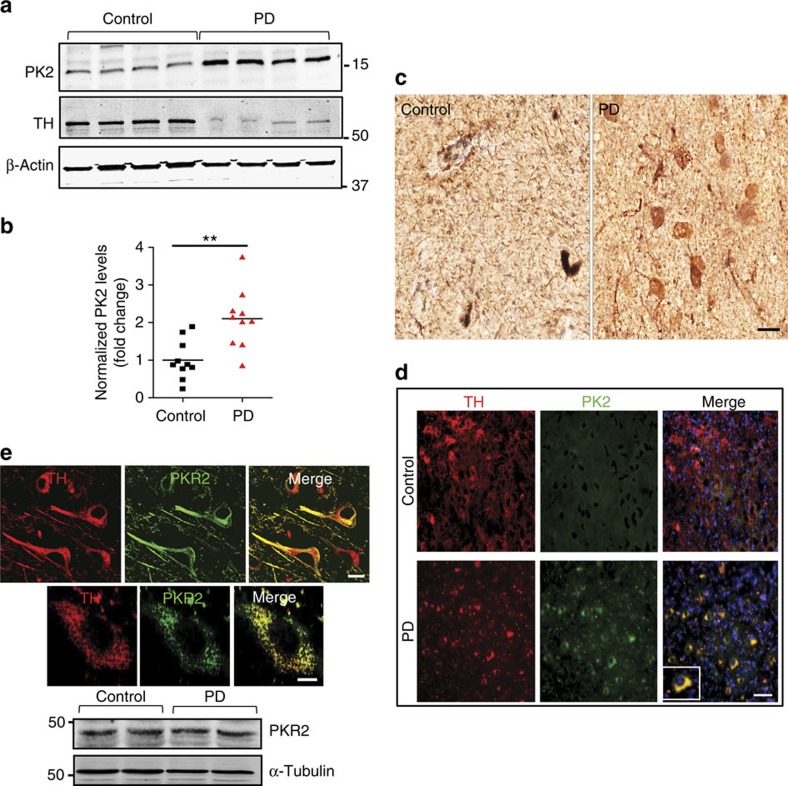
PK2 is elevated in the substantia nigra of human PD patients. (**a**) Representative western blot for PK2 expression in nigral lysates from PD patients and age-matched controls probed using a rabbit polyclonal antibody directed against the human PK2 protein. Increased levels of PK2 were evident in PD patient samples compared with control subjects. A representative TH western blot is also shown. (**b**) Densitometric band intensities for PK2, determined from 10 control and 10 PD samples normalized using β-Actin as the internal control. Data represented as mean±s.e.m. and expressed as fold change over control with *n*=10. Asterisks denote a significant (^**^P<0.01) difference between control and PD samples using Student's *t*-test. (**c**) IHC for PK2 in human nigral sections using DAB immunostaining showed increased neuronal PK2 expression in PD brains. Scale bar, 40 μm. (**d**) Double-labelling immunofluorescence for PK2 (green), TH (red) and nuclei (blue). Scale bar, 40 μm. Representative images from one of the three experiments are shown. (**e**) Top: double-labelling IHC shows expression of PKR2 (green) on human nigral TH^+^ (red) neurons. The top panel is × 60 magnification to show PKR2 merging with several TH-positive neurons (scale bar, 20 μm), and the bottom panel is × 63 magnification with × 2.5 zoom to get a high magnification image of PKR2 in one TH-positive cell (scale bar, 10 μm). Bottom: western blots for PKR2 expression in nigral lysates from control and PD brains (three cases each) revealed that PKR2 was highly expressed in control brains and its expression levels did not change in PD patients. A representative TH western blot is also shown.

Nigral tissue lysates from the post-mortem brain samples of PD patients (Supplementary Table 2) and age-matched controls were probed for PK2 protein expression by western blotting. Increased PK2 expression was evident in PD brains as compared with age-matched control brains (Fig. 3a and Supplementary Fig. 4A). Densitometric analysis of PK2 band intensities from 10 controls and 10 PD samples indicated that PK2 levels in PD brain lysates were about two-fold higher (Fig. 3b). For reasons not clear at present, in contrast to mouse brains, we observed a small amount of PK2 in control human brains. Perhaps a higher basal level of PK2 expression occurs in human brains compared with mice, or the effect is age-related. We also confirmed increased neuronal PK2 expression in DAB-stained post-mortem nigral tissue sections obtained from PD patients relative to age-matched controls (Fig. 3c). PK2 and TH co-labelling (Fig. 3d) revealed that PK2 was largely absent on TH^+^ dopaminergic neurons in control brain sections. However, in PD brain sections, intense and specific PK2 staining was localized to surviving TH^+^ dopaminergic neurons. Together, these results demonstrate for the first time that PK2 expression is elevated in PD patients, providing clinical relevance to our findings from experimental PD models.

We also confirmed that the PKR2 receptor, measured by IHC and immunoblot analysis, is expressed on dopaminergic neurons in the human brain (Fig. 3e and Supplementary Fig. 4B) using nigral samples from control and PD cases. Again, constitutive PKR2 expression showed no significant difference between control and diseased patient samples. Although PKR2 is constitutively expressed on neurons across different brain regions^14^,^15^, a significant decrease in PKR2 expression is not evident in nigral lysates of PD brains perhaps because it is probable that this protein might be expressed in other neuronal phenotypes at substantial levels. Supplementary Fig. 5 shows that PKR2 is expressed in many MAP2-positive neurons in human SN samples. These results confirm that secreted PK2 can act directly on dopaminergic neurons via the constitutively expressed PKR2 receptor.

### PK2 reduces MPP^+^-induced dopaminergic neuronal cell death

**Figure 4:**
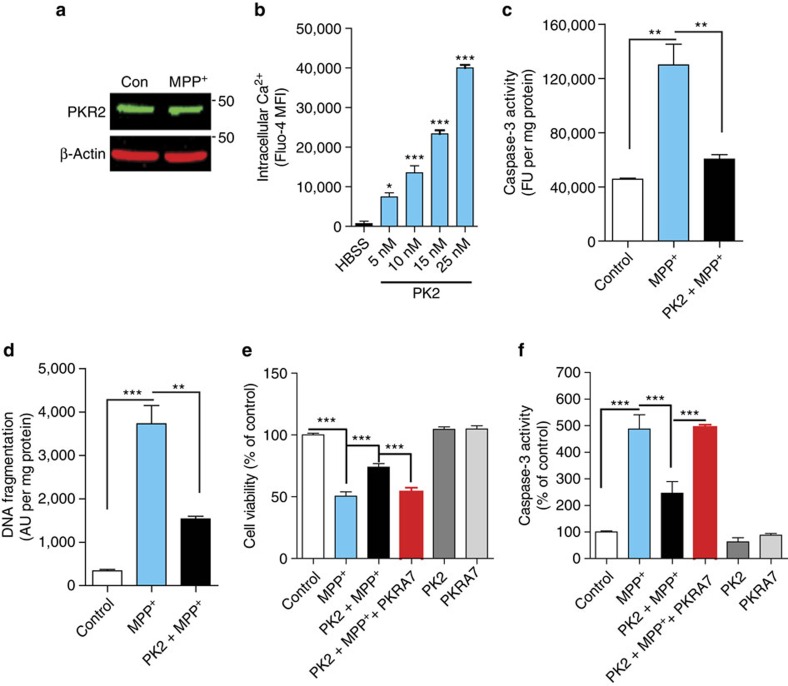
Recombinant PK2 protects dopaminergic N27 cells against MPP^+^-induced dopaminergic neuronal cell death. (**a**) Representative western blot from one of the three experiments showing no changes in PKR2 expression during MPP^+^-induced cell death in N27 dopaminergic cells. (**b**) Calcium mobilization (flux) induced by rPK2 in N27 cells, measured as mean fluorescent intensity (MFI) of Fluo-4. Calcium-free HBSS was used on the control group (one-way ANOVA with Bonferroni post-test comparison and *n*=3). (**c**,**d**) Exogenously stimulated PK2 signalling attenuated caspase-3 activation induced by MPP^+^ at 8 h (**c**) and DNA fragmentation at 16 h after MPP^+^ treatment (**d**). N27 cells were treated with MPP^+^ (200 μM) or co-treated with PK2 (25 nM) and cells were collected for assays of caspase-3 activity at 8 h and DNA fragmentation at 16 h. The data were expressed as fluorescence units (FU) per mg of protein for caspase-3 activity and absorbance units (AU) per mg protein for DNA fragmentation (one-way ANOVA with Bonferroni post-test comparison and *n*=3). (**e**,**f**) Co-treatment with the specific PK2 receptor antagonist PKRA7 blocked PK2's neuroprotection in MTS (**e**) (one-way ANOVA with Bonferroni post-test comparison and *n*=4–8) and caspase-3 activity (**f**) (one-way ANOVA with Bonferroni post-test comparison and *n*=3) assays. N27 cells were treated with 200 μM MPP^+^ for 24 h in the presence or absence of 25 nM PK2 and 2 μM PKRA7. Data represented by group mean±s.e.m. (*P<0.05, ^**^P<0.01 and ^***^P<0.001).

**Figure 5:**
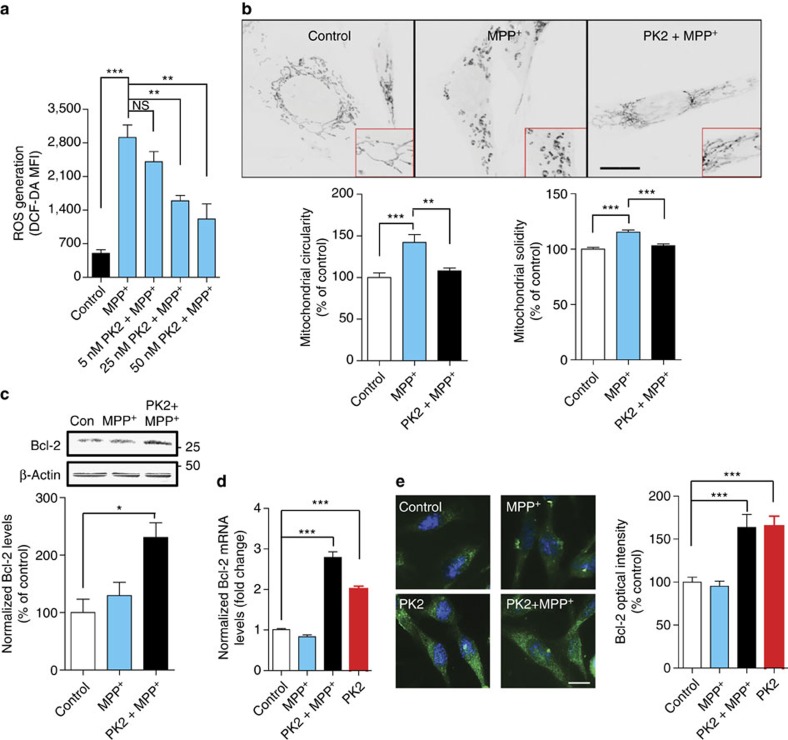
Recombinant PK2 protects dopaminergic N27 cells against MPP^+^-mediated oxidative stress and mitochondrial dysfunction. (**a**) Co-treatment with nanomolar doses of rPK2 attenuated MPP^+^-induced ROS generation in a dose-dependent manner. N27 cells were treated with 300 μM MPP^+^ for 8 h and total iROS was determined by quantifying the DCF-DA mean fluorescent intensity (MFI) using a plate reader with a total *n*=3 (NS=non-significant, *P*=0.4939). (**b**) PK2 signalling blocked MPP^+^-induced mitochondrial fragmentation. Top: N27 cells were treated with MPP^+^ (300 μM) for 16 h and incubated with MitoTracker Red dye to visualize mitochondria. Scale bar, 20 μm. Bottom: morphometric analysis (ImageJ plug-in^55^) of mitochondrial structures showed that PK2 co-treatment significantly reduced MPP^+^-induced mitochondrial circularity (left) and solidity (right) with *n*=8–10. (**c**–**e**) PK2 upregulates anti-apoptotic protein Bcl2. Bcl-2 protein (*n*=2–3) (**c**) and mRNA expression (*n*=3–15) (**d**) were determined by western blotting and qPCR, respectively. PK2 treatment upregulated the basal level of Bcl-2 expression, which remained elevated during treatment with MPP^+^. Bcl-2 expression was also visualized by immunofluorescence (*n*=3) (**e**). Scale bar, 10 μm. Data represented by group mean±s.e.m. (*P<0.05 and ^***^P<0.001). All statistics for this figure were determined using one-way ANOVA with Bonferroni post-test comparison.

**Figure 6:**
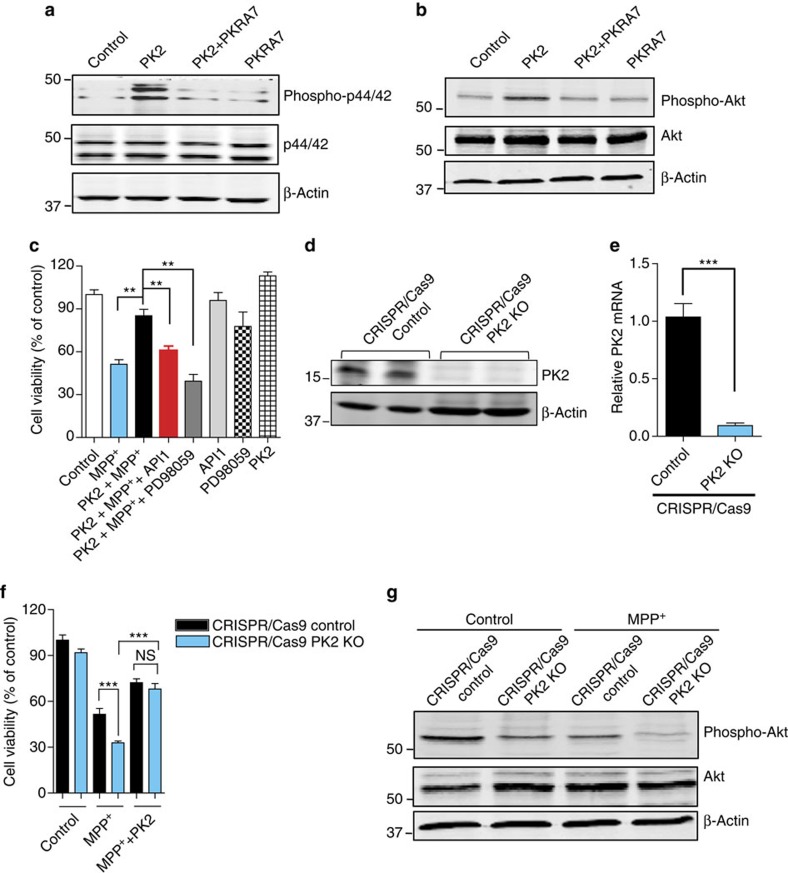
Recombinant PK2 (rPK2) activates Akt and ERK (p44/42) signalling pathways to protect N27 cells from MPP^+^ toxicity. (**a**,**b**) N27 cells were untreated or treated with 25 nM rPK2 in the absence or presence of 2 μM PKRA7 for 3 h. Cells were collected and subjected to western blot analysis of ERK (**a**) and Akt (**b**) activation. (**c**) N27 cells were untreated or treated with 300 μM MPP^+^ in the absence or presence of 25 nM rPK2, 100 nM Akt inhibitor API1 or 50 μM ERK inhibitor PD98059 for 24 h. Cell viability was then measured by MTS assay. Data are expressed as the mean±s.e.m. of three independent experiments using one-way ANOVA with Bonferroni post-test comparison with *n*=7–22. (**d**,**e**) CRISPR/Cas9-based knockdown of PK2 in N27 cells infected with CRISPR/Cas9 PK2 KO lentivirus or CRISPR/Cas9 control non-target lentivirus. PK2 expression was analysed by western blot (**d**) and real-time RT–PCR (**e**) (Student's *t*-test with *n*=6). (**f**,**g**) CRISPR/Cas9 PK2 KO or CRISPR/Cas9 control lentivirus-infected N27 cells were treated with or without 300 μM MPP^+^ in the presence or absence of 25 nM rPK2. Cell viability was analysed by MTS assay (**f**) (one-way ANOVA with Bonferroni post-test comparison and *n*=8; NS, non-significant, *P*=0.9065) and Akt activation was measured by western blot (**g**) 24 h after MPP^+^ treatment. Asterisks denote statistical significance (*P<0.05, ^**^P<0.01 and ^***^P<0.001. NS, non-significant).

We next identified the functional role of secreted PK2 during dopaminergic cell death. We found that PKR2 was highly expressed on N27 dopaminergic cells, and consistent with our *in vivo* findings, its expression did not change with MPP^+^-induced degeneration (Fig. 4a). We also confirmed that rPK2 treatment mobilized calcium in these cells (Fig. 4b), indicating that functional PK2 receptors are present on dopaminergic neuronal cells. When we treated N27 dopaminergic cells with 200 μM MPP^+^ in the presence or absence of nanomolar doses of rPK2, the rPK2 (25 nM) co-treatment significantly reduced MPP^+^-induced activation of caspase-3 and DNA fragmentation measured at 8 and 16 h time points, respectively (Fig. 4c,d), suggesting that PK2 is protective in dopaminergic cells during neuronal cell death. Furthermore, co-treatment with a specific PK2 receptor antagonist, PKRA7 (ref. 22), effectively blocked the neuroprotective action of rPK2 (Fig. 4e,f). We then showed that co-treatment with 5–50 nM rPK2 mitigated MPP^+^-induced intracellular ROS levels in a dose-dependent manner (Fig. 5a). Next, we visualized mitochondrial integrity by using MitoTracker Red fluorescent dye in MPP^+^-treated N27 cells in the presence and absence of rPK2. MPP^+^ caused considerable fragmentation of mitochondria compared with untreated controls, whereas in rPK2 co-treated cells, mitochondrial fragmentation was significantly reduced (Fig. 5b, top panel). These changes were further evidenced by quantifying key morphometric parameters including mitochondrial circularity and solidity (Fig. 5b, bottom panels). To understand the mechanism by which PK2 signalling reduces mitochondrial dysfunction in dopaminergic cells, we examined the relevant protective signalling pathways. Both rPK2 co-treatment with MPP^+^ and rPK2-alone significantly increased mRNA and protein levels of the mitochondrial anti-apoptotic protein Bcl-2 in N27 cells (Fig. 5c–e). To further investigate PK2's neuroprotection in greater mechanistic detail, we evaluated the contribution of key downstream effectors of PK2 signalling in other systems, including ERKs (p44/42) and Akt^23^,^24^,^25^,^26^. In N27 dopaminergic cells, phosphorylation of Akt and ERKs was observed 3 h after rPK2 stimulation (Fig. 6a,b), confirming that these pathways are activated downstream of PK2 signalling. The PK2-stimulated Akt and ERK activations were both completely blocked by the PK2 receptor antagonist PKRA7 (ref. 22), indicating the specificity of PK2 signalling in activating these kinases. The ERK-specific inhibitor PD98059 (ref. 27) significantly attenuated the protection from MPP^+^-induced cell death afforded by rPK2 treatment (Fig. 6c), whereas PD98059-alone had no effect on cell viability. Similar results were obtained with the Akt inhibitor API-1 (ref. 28) (Fig. 6c). These results indicate that the neuroprotective effect of rPK2 was mediated, at least in part, by activating Akt and ERK/MAPK signalling pathways. Finally, we tested whether a genetic knockdown of endogenous PK2 sensitizes N27 cells to MPP^+^-induced toxicity. Transduction of N27 cells with the CRISPR/Cas9-based PK2 KO lentivirus almost completely abolished PK2 protein and mRNA expression in N27 cells (Fig. 6d,e). Our cytotoxicity results demonstrated that PK2 deficiency via CRISPR/Cas9 indeed sensitized the cells to MPP^+^ toxicity, and that the addition of 25 nM rPK2 reversed this sensitivity (Fig. 6f). Knockdown of PK2 significantly reduced Akt activity in both untreated and MPP^+^-treated N27 cells (Fig. 6g). Together, these results demonstrate for the first time that PK2 signalling protects dopaminergic cells against MPP^+^-mediated oxidative stress, mitochondrial dysfunction and cell death by activating ERK and Akt signalling pathways, implying that PK2 secreted from dopaminergic neurons is a compensatory protective factor acting locally against neuronal death.

### PK2 overexpression promotes mitochondrial biogenesis

**Figure 7:**
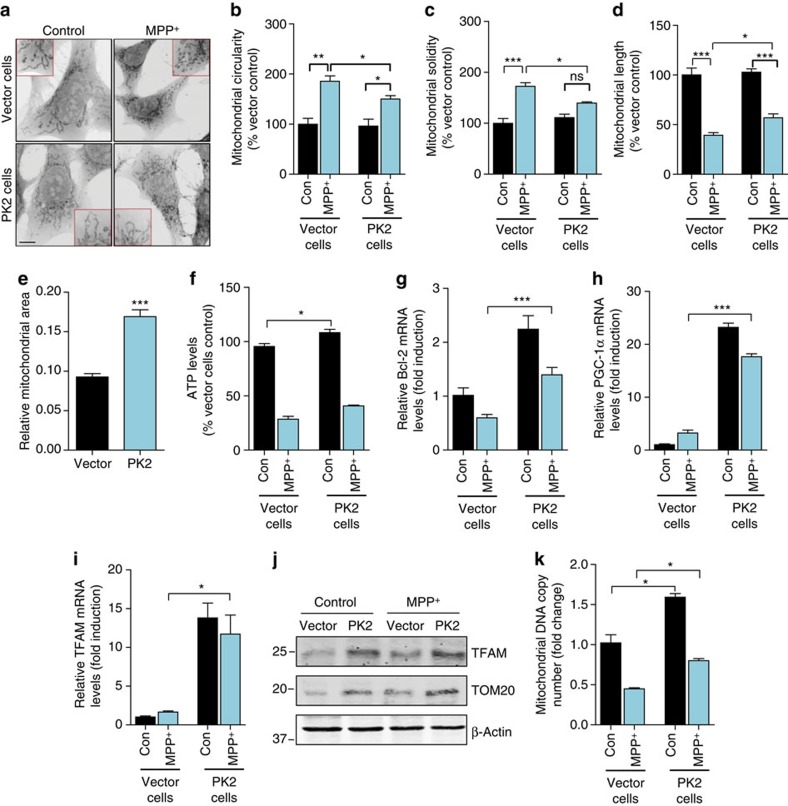
Stable expression of PK2 protects against MPP^+^-mediated mitochondrial dysfunction and promotes mitochondrial biogenesis. (**a**-**d**) Reduced mitochondrial fragmentation in PK2-overexpressing cells. Mitochondrial fragmentation was visualized using MitoTracker Red in vector and PK2-overexpressing cells treated with MPP^+^ (300 μM) for 16 h. Scale bar, 10 μm. Reduced mitochondrial circularity (**b**), solidity (**c**) and increased mitochondrial length (**d**) were evident in cells overexpressing PK2 (Student's *t*-test with *n*=3; NS, non-significant, *P* value=0.0767 in **c**). (**e**) Increased mitochondrial area in PK2-overexpressing cells. Mitochondrial area was measured by thresholding the MitoTracker Red images to compare with the total area of the cell. PK2-overexpressing cells had significantly more mitochondrial area/total cell area compared with vector cells (Student's *t*-test with *n*=6–7). (**f**) Overexpression of PK2 increased ATP levels. PK2-overexpressing and vector cells were exposed to 300 μM MPP^+^ for 16 h, and the amount of cellular ATP produced was measured using the Luminescent Cell Viability assay kit (one-way ANOVA with Bonferroni post-test comparison and *n*=4). (**g**–**k**) Overexpression of PK2 upregulated mitochondrial biogenesis pathways. Real-time qPCR analysis of Bcl-2 (**g**) (Student's *t*-test with *n*=3), PGC-1α (**h**) (one-way ANOVA with Bonferroni post-test comparison and *n*=3) and TFAM (**i**) (one-way ANOVA with Bonferroni post-test comparison and *n*=3–6) shows increased mRNA expression of Bcl-2, PGC-1α and TFAM in PK2-overexpressing cells compared with vector control cells 24 h post MPP^+^ (300 μM) treatment. Increased TFAM and TOM20 protein levels were found in PK2 cells compared with vector cells 24 h post MPP^+^ (300 μM) (**j**). Analysis of mitochondrial ND1 as a marker of mitochondrial DNA content shows increased mitochondrial DNA copy number (**k**) in PK2-overexpressing cells compared with vector control cells exposed to vehicle or 300 μM MPP^+^ for 24 h (one-way ANOVA with Bonferroni post-test comparison and *n*=6). Data represented by group mean±s.e.m. (*P<0.05, ^**^P<0.01 and ^***^P<0.001, relative to relevant control or MPP^+^-treated groups).

Having confirmed that exogenous rPK2 is neuroprotective, we next tested endogenous PK2 overexpression. We engineered dopaminergic MN9D cells to express human wild-type PK2 by stably transfecting them with pCMV6-PK2-myc expression plasmid or control vector. Stable PK2 overexpression was validated by western blotting using the PK2 antibody (Supplementary Fig. 6A) and immunostaining for PK2-myc using a myc-tag antibody (Supplementary Fig. 6B). The basal levels of PKR2 receptor in these cells were unaffected (Supplementary Fig. 6A). After vector and PK2-overexpressing cells were treated with MPP^+^ (300 μM), we confirmed that, similar to rPK2 treatment, PK2-expressing cells were significantly protected against MPP^+^ neurotoxicity compared with vector control cells (Supplementary Fig. 6C–E). This protective effect of PK2 was attenuated by co-treating with 2 μM PKRA7. To investigate whether stable PK2 expression protects against MPP^+^-induced mitochondrial dysfunction, we first performed MitoTracker Red imaging in an N27 cell line stably expressing human PK2, established by a lentivirus-based gene delivery method. MPP^+^ caused mitochondrial fragmentation in N27 vector control cells, as evidenced by donut-shaped morphology (Fig. 7a). However, in MPP^+^-treated PK2-expressing cells, longer strand-like mitochondria were preserved. Quantification of mitochondrial circularity, solidity and length confirmed these findings (Fig. 7b–d). The fluorescent signals from MitoTracker Red (Fig. 7e) revealed a larger mitochondria area in PK2-expressing cells relative to vector cells, suggesting a role for PK2 in regulating mitochondrial biogenesis. We also examined whether PK2 protected against MPP^+^-induced cellular ATP depletion, which could result from mitochondrial dysfunction. PK2-expressing and vector control cells were treated with or without MPP^+^ for 16 h. PK2 overexpression increased basal levels of ATP production; however no significant differences were observed between MPP^+^-treated PK2-expressing and vector control cells (Fig. 7f). Consistent with our rPK2 treatment results, stable PK2 overexpression also increased basal levels of Bcl-2 mRNA, and it mitigated the loss of Bcl-2 caused by MPP^+^ treatment relative to vector control cells (Fig. 7g). Furthermore, the transcription of PGC-1α (Fig. 7h) and TFAM (Fig. 7i), key genes controlling mitochondrial biogenesis, were both markedly upregulated in PK2-expressing cells compared with vector cells. In line with these results, stably expressing PK2 increased protein levels of TFAM as well as a mitochondria marker, mitochondrial import receptor subunit TOM20 (Fig. 7j and Supplementary Fig. 7). Finally, PK2 signalling increased the mitochondrial DNA copy number in both control and MPP^+^-treated N27 cells (Fig. 7k). Together, these data demonstrate for the first time that PK2 protects dopaminergic cells by activating mitochondrial biogenesis pathways. These findings also establish an exciting new link between PK2 signalling and the PGC-1α pathway as the mechanism by which PK2 mediates protection against mitochondrial dysfunction and death in dopaminergic cells.

### PK2 reduces primary DAergic neuronal loss induced by MPP^+^

**Figure 8:**
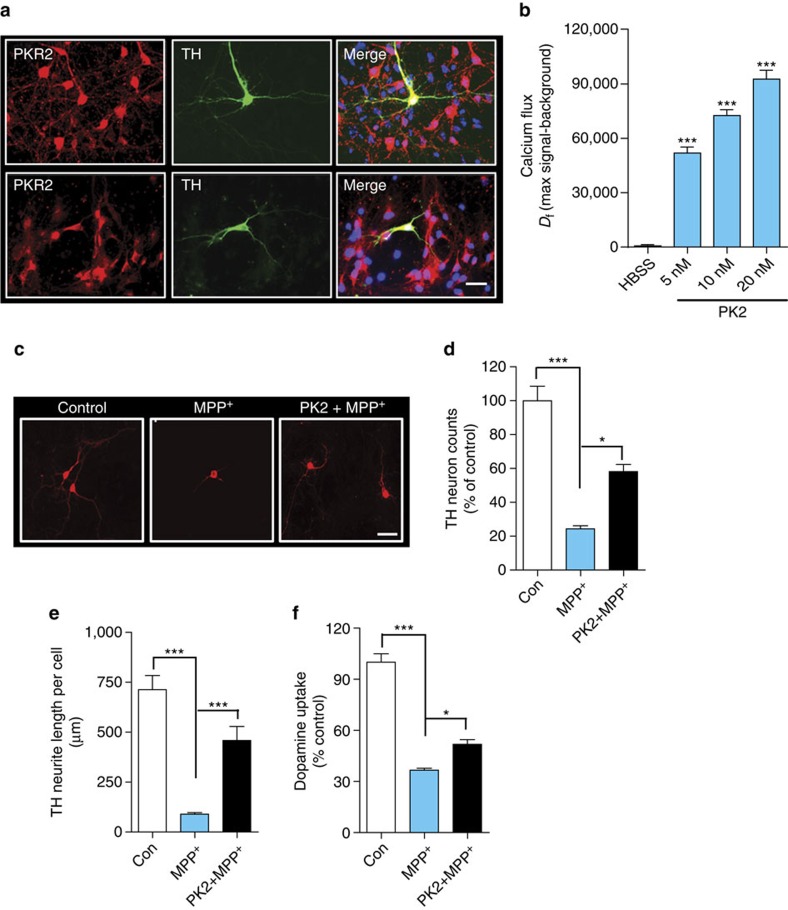
Recombinant PK2 protects primary dopaminergic neurons from MPP^+^ neurotoxicity. (**a**) Expression of prokineticin receptors on primary dopaminergic neurons. Primary mesencephalic neuron cultures were stained for PKR1 and PKR2 (red), and TH (green). Both PKR1 and PKR2 were expressed on TH^+^ dopaminergic neurons. Scale bar, 10 μm. (**b**) Calcium mobilization by rPK2 in primary mesencephalic neuron cultures. Primary neuron cultures were treated with nanomolar doses of rPK2 and the calcium flux was quantified via Fluo-4 NW calcium assay. Control cells were treated with calcium-free HBSS containing PK2. Calcium flux was expressed as the net change in fluorescence signal (*D*_f_) represented as the maximum signal after baseline subtraction for each dose of PK2. Calcium was mobilized by PK2 in a dose-dependent manner. Data represented by group mean±s.e.m. Asterisks denote significant (^***^P<0.001) differences between HBSS-treated control and PK2-treated groups. (**c**) Immunocytochemistry for TH^+^ dopaminergic neurons in EVM cultures treated with MPP^+^ and PK2. The PK2-treated cultures significantly improved dopaminergic neuron survival and function compared with MPP^+^-alone, showing less degeneration of dopaminergic cell bodies and neurites. Representative images of dopaminergic neurons in primary EVM cultures were acquired at 20 × using a Nikon TE2000-U fluorescence microscope. MPP^+^ caused extensive degeneration of dopaminergic neurons, which was attenuated by rPK2. Scale bar, 10 μm. (**d**–**f**) TH^+^ neuron counts (**d**) (one-way ANOVA with Bonferroni post-test comparison and *n*=4), TH^+^ neurite length measurements (**e**) (one-way ANOVA with Bonferroni post-test comparison and *n*=3) and dopamine uptake functional assay (**f**) (Student's *t*-test with *n*=3) in primary EVM neuronal cultures. Primary EVM cultures were treated with MPP^+^ (5 μM) for 48 h or co-treated with rPK2 (25 nM), which was added initially and again 24 h later. Data represented by group mean±s.e.m. (*P<0.05 and ^***^P<0.001).

Before testing if PK2 protects primary dopaminergic neurons from MPP^+^-induced neurodegeneration, we verified that primary dopaminergic neurons expressed both PKR1 and PKR2 (Fig. 8a), and rPK2-induced calcium mobilization confirmed that these receptors were functionally active (Fig. 8b). Given the constitutive expression of PKR2 in various neuronal sub-populations, the observed calcium changes are not specific for dopaminergic cells, which compose about 5% of the total cells in this population. We then treated primary mesencephalic neuronal cultures for 48 h with 5 μM MPP^+^-alone or co-treated with 25 nM rPK2, which was added again 24 h later. MPP^+^ reduced the number of TH^+^ dopaminergic neurons to almost 30% of the vehicle-treated group, indicating extensive dopaminergic degeneration. PK2 treatment significantly (*P<0.05 versus MPP^+^-alone) mitigated MPP^+^-induced dopaminergic degeneration (Fig. 8c,d). MPP^+^ induced a dramatic decrease (∼85%) in neurite length of TH-immunoreactive neurons, which was significantly improved by rPK2 treatment (Fig. 8c,e). The functional activity of surviving primary dopaminergic neurons was confirmed by dopamine uptake assays. PK2 significantly ameliorated the MPP^+^-induced loss of dopamine uptake activity (Fig. 8f). Together, these results further corroborate a novel neuroprotective role for PK2 in dopaminergic neurons *in vitro*.

### PKRA7 increases susceptibility to MPTP toxicity in mice

**Figure 9:**
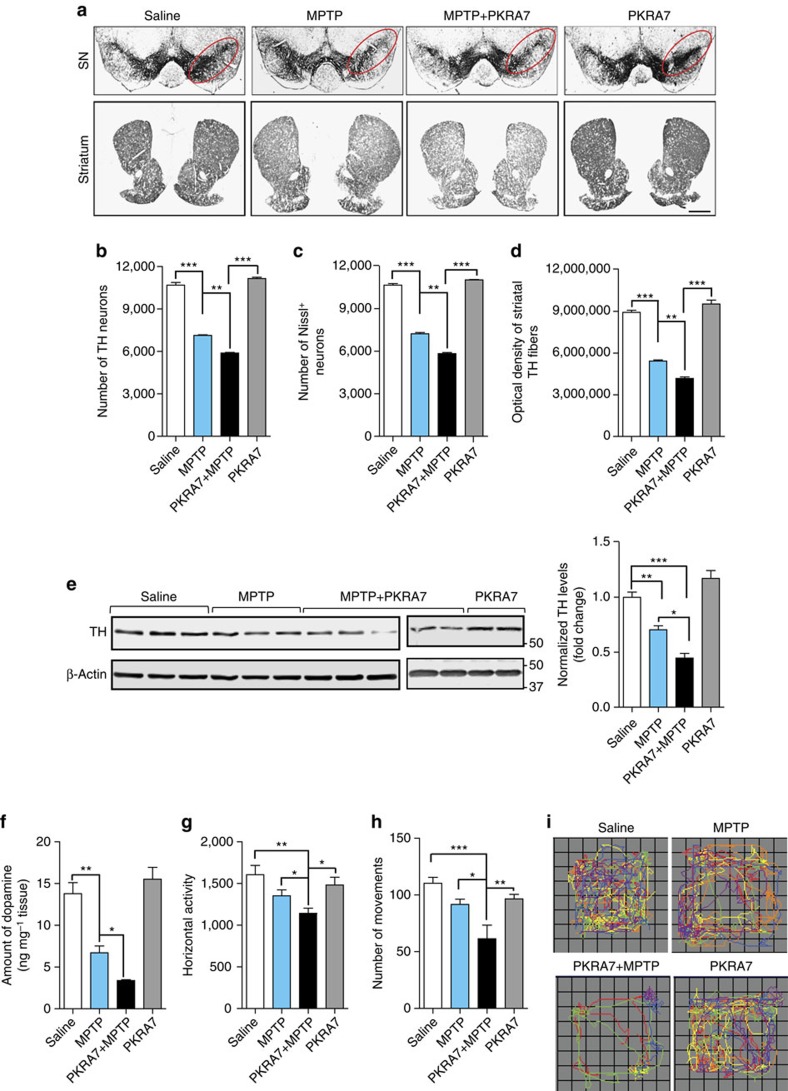
Inhibition of endogenous PK2 signalling by a receptor blocker exacerbates MPTP-induced toxicity in mice. Mice were intraperitoneally injected with 18 mg kg^−1^ MPTP or equal volumes of saline (vehicle), once daily for three consecutive days. PKRA7 (20 mg kg^−1^ per day) was given i.p. 24 h before MPTP treatment and continued once daily for 10 consecutive days until euthanization. (**a**) TH-DAB immunostaining in the substantia nigra (SN) and striatum (× 2 magnification). Scale bar, 200 μm. (**b**,**c**) Stereological counts of TH-positive (**b**) and Nissl-positive (**c**) neurons in the SN of ventral midbrain. (**d**) Integrated density quantification of DAB immunostaining in the striatum. Data represented by group mean±s.e.m. of three mice per group using one-way ANOVA with Bonferroni post-test comparison and *n*=3 (^**^P<0.01 and ^***^P<0.001, relative to relevant control or MPTP-treatment groups). (**e**) Western blot analysis of SN samples probed for TH and its quantification (right panel) show reduced TH levels when PKRA7 is administered along with MPTP. Data represented by group mean±s.e.m. using one-way ANOVA with Bonferroni post-test comparison and five to seven mice per group (*P<0.05, ^**^P<0.01 and ^***^P<0.001, relative to control or MPTP-treated groups). (**f**) Compared with MPTP-alone, PKRA7 treatment significantly reduced striatal dopamine levels, as measured using high-performance liquid chromatography (HPLC). Data represented by group mean±s.e.m. using Student's *t*-test with *n*=2–3 mice per group (*P<0.05, ^**^P<0.01, and ^***^P<0.001, relative to relevant control or MPTP-treatment groups). (**g**–**i**) Mice were tested for motor activities 7 days after the last dose of MPTP. (**i**) Representative movement tracks of mice are shown. Behaviour analysis showing horizontal activity (**g**) (Student's *t*-test with *n*=7) and number of movements (**h**). Data represented as the mean±s.e.m. using one-way ANOVA with Bonferroni post-test comparison and *n*=7–8 mice per group (*P<0.05, ^**^P<0.01).

We utilized the small-molecule PK2 receptor antagonist PKRA7 (ref. 22) to block PK2 signalling in the MPTP model of PD because knockout mice for PK2 and its receptors show significant defects in brain development and architecture, potentially involving dopamine pathways that would confound our studies^1^,^3^,^5^,^6^. Since the acute MPTP treatment paradigm causes severe loss of dopaminergic neurons, it is not amenable to testing the effect of PK2 receptor antagonists, which we anticipated would exacerbate neuronal loss. Therefore, we adopted a subacute MPTP treatment paradigm that results in partial nigral dopaminergic degeneration^29^,^30^,^31^. Furthermore, the time course of neurodegeneration in the sub-acute model is linear and well characterized, therefore the dose and treatment regimen can be tailored to achieve the partial dopaminergic degeneration required to study the effect of blocking PK2 signalling. Once-daily injections of MPTP (18 mg kg^−1^ per day, i.p.) were administered for three consecutive days to induce moderate neurochemical and behavioural deficits. One day before the first MPTP treatment, we initiated once-daily i.p. injections of PKRA7 (20 mg kg^−1^ per day) for 10 days. TH immunostaining of coronal sections from the SN and striatum showed that while PKRA7-alone had no effect on the nigrostriatal dopaminergic system, it augmented MPTP-induced neurodegeneration in both the striatum and SN (Fig. 9a). These findings were confirmed by stereological quantification of TH^+^ neurons and Nissl-positive cells in the SN as well as densitometry of the striatal TH innervation (Fig. 9b–d). We found that the number of Nissl-positive total neurons did not significantly differ from that of TH^+^ neurons, which is consistent with previous findings^32^,^33^. Western blotting of nigral tissue lysates showed moderately reduced TH expression in the SN of MPTP-treated mice when compared with control mice, whereas PKRA7 significantly enhanced nigral TH loss in MPTP-treated mice (Fig. 9e). In addition, PKRA7 did not alter PK2 and PKR2 levels in the SN of either PKRA7-alone or PKRA7/MPTP-treated mice compared with saline and MPTP-treated mice (Supplementary Fig. 8). Nigral PK2 levels did not differ significantly between vehicle- or MPTP-injected mice after 7 days (Supplementary Fig. 8). This result is not surprising since we have observed that the MPTP-induced PK2 expression started to decrease by the third day post-MPTP injection (Fig. 2a). As expected, three doses of MPTP produced a ∼51% loss of striatal dopamine compared with vehicle-treated control mice as measured by HPLC at day 7 after last administration of saline or MPTP, a more sensitive and precise measure of MPTP-induced dopaminergic degeneration. However, PKRA7 treatment significantly exacerbated MPTP-induced dopamine depletion by ∼75% compared to saline, whereas PKRA7-alone had no effect on striatal dopamine levels (Fig. 9f). In line with the histological, biochemical and neurochemical evidence, both the representative locomotor activity maps of individual open-field movements (Fig. 9i) and the group averages of horizontal activity and total number of movements (Fig. 9g,h) indicated that PKRA7 worsens the behavioural deficits of MPTP-treated mice. Together, our results from a preclinical model of PD further support that blocking endogenous PK2 signalling exacerbates dopaminergic degeneration, resulting in increased neuropathology and behavioural deficits.

### rAAV-mediated PK2 overexpression is neuroprotective in mice

**Figure 10:**
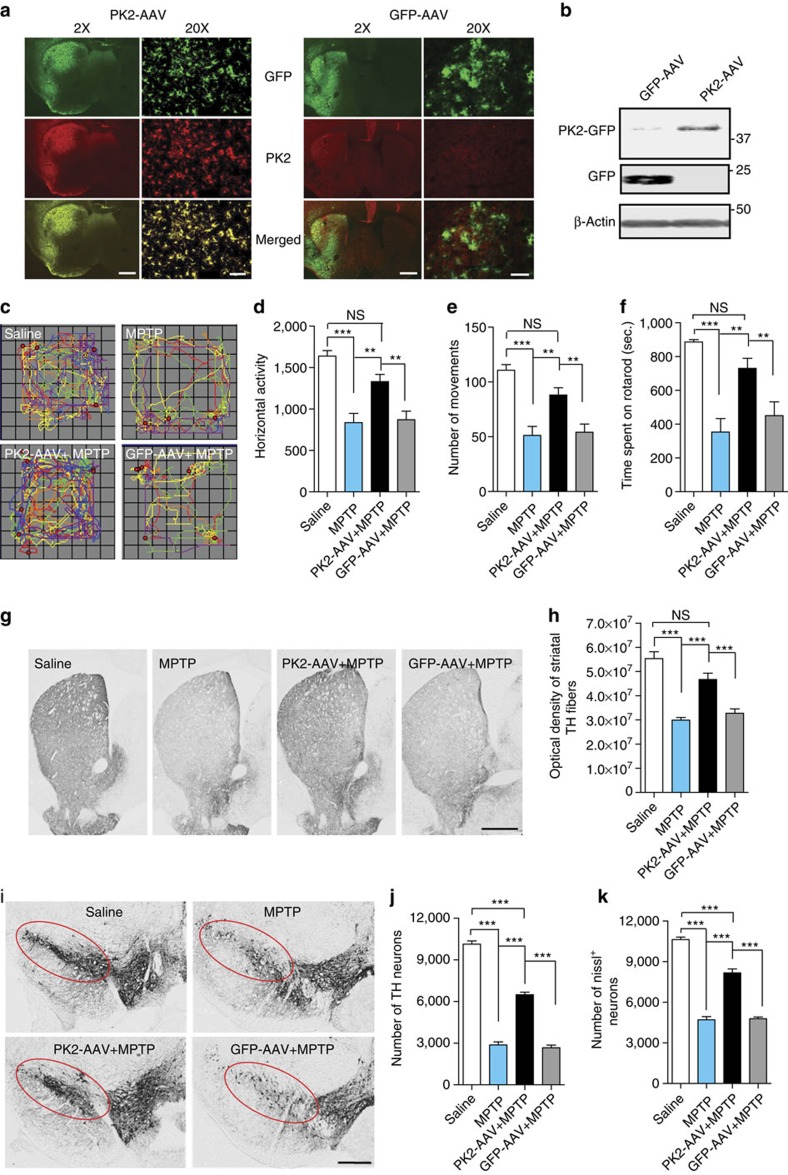
PK2 overexpression protects against MPTP-induced neurotoxicity in mice. Mice were stereotaxically injected into the striatum with either GFP-AAV or GFP-tagged PK2-AAV and allowed to reach maximal viral expression for 4 weeks. The mice were then intraperitoneally injected with 20 mg kg^−1^ MPTP or equal volumes of saline (vehicle), once daily for four consecutive days and euthanized 24 h after the last injection. (**a**) Expression of PK2 virus in the striatum by immunofluorescence microscopy. Brain sections were probed with anti-GFP (green) and anti-PK2 (red) antibodies and imaged to show that the two proteins co-localize together (× 2 and × 20 images). Scale bar, 200 μm (× 2 images); 40 μm (× 20 images). (**b**) PK2 overexpression levels by western blotting. Striatal protein lysates probed for either GFP or PK2 show that the PK2 virus was able to overexpress PK2 in the striatum. Since GFP was directly fused to PK2, we observed only one band corresponding to the tagged PK2-GFP fusion protein and did not detect a separate 25-kDa GFP band in the GFP-tagged PK2-AAV samples. (**c**–**f**) Mice were tested for motor activities 1 day after the last dose of MPTP. (**c**) Representative VersaPlot movement tracks of mice are shown. Behavioural data showing horizontal activity (**d**), number of movements (**e**) and rotarod activity (**f**). Data represented by group mean±s.e.m. using one-way ANOVA with Bonferroni post-test comparison and *n*=12–24 mice per group (^**^P<0.01 and ^***^P<0.001, relative to relevant control or MPTP-treated groups; NS, non-significant, *P*=0.0912 (**d**), 0.1048 (**e**), 0.55 (**f**) and 0.054 (**h**)). (**g**–**j**) PK2 AAV protects against MPTP-induced loss of dopaminergic neurons. (**g**) TH-DAB immunostaining in the striatum (× 2 magnification). Scale bar, 200 μm. (**h**) Integrated density quantification of DAB immunostaining in the striatum (one-way ANOVA with Bonferroni post-test comparison and *n*=12–16). (**i**) TH-DAB immunostaining (× 2 magnification) in the substantia nigra (SN). Scale bar, 100 μm. (**j**–**k**) Stereological counts of TH-positive (**j**) and Nissl-positive (**k**) neurons in the SN of the ventral midbrain. Data represented by group mean±s.e.m. using one-way ANOVA with Bonferroni post-test comparison and *n*=3 mice per group (^***^P<0.001, relative to relevant control or MPTP-treated groups).

To further demonstrate the neuroprotective action of PK2 *in vivo*, we investigated whether recombinant adeno-associated virus (rAAV) 2/5-mediated PK2 overexpression confers neuroprotection against MPTP in mice. Recombinant AAVs encoding GFP-tagged human PK2 or GFP-alone were stereotaxically injected into the striatum of C57BL/6 mice. Striatal delivery of neuroprotective growth factors with an rAAV gene delivery system has been more effective than nigral delivery^34^,^35^. Four weeks after the virus injection, we performed GFP/PK2 double-labelling IHC, confirming that rAAV-mediated delivery of GFP-PK2 or GFP resulted in widespread and efficient transgene expression in the striatum (Fig. 10a). Consistent with the initial observation of low basal PK2 expression in the striatum, only marginal endogenous PK2 immunoreactivity was observed in the striatum of AAV-GFP-transduced mice. We confirmed GFP-PK2 or GFP transgene expression in AAV-injected mice by western blotting striatal lysates with a hamster monoclonal anti-PK2 (top panel) or anti-GFP (middle panel) antibody (Fig. 10b). GFP-PK2-AAV expression was lower than that of GFP-AAV (Fig. 10b), possibly because PK2 is a secreted protein, and therefore, GFP-tagged PK2 can be released from GFP-PK2-AAV-infected cells. To confirm the retrograde transport of PK2 from the striatum to the SN, we determined PK2 expression in the SN from animals that received viral gene delivery in the striatum. IHC revealed GFP-PK2 co-labelled in TH^+^ neurons of the SN, indicating retrograde transport of the GFP-PK2 transgene from the striatum to the SN (Supplementary Fig. 9). Four weeks after virus injection, mice were exposed to a sub-acute MPTP paradigm, treating mice with 20 mg kg^−1^ MPTP daily for 4 days to produce significant dopaminergic neurodegeneration in the SN and striatum; the subacute paradigm is routinely used to assess the efficacy of putative neuroprotective agents^36^,^37^. Saline- and MPTP-alone groups were also included. Spontaneous open-field movements lasting 10 min were recorded 24 h after the last MPTP dose and individual track plots were generated for each mouse (Fig. 10c). The locomotor activity of AAV-PK2-transduced mice treated with MPTP was much improved relative to MPTP-alone and AAV-GFP/MPTP groups (Fig. 10d,e). Similarly, AAV-PK2 transduction significantly restored the 20-r.p.m. rotarod performance of MPTP-treated mice (Fig. 10f). Based on these results, we conclude that AAV-mediated PK2 overexpression reverses MPTP-induced motor deficits. Furthermore, PK2 overexpression protected against MPTP-induced dopaminergic terminal loss in the striatum (Fig. 10g,h). TH^+^ DAB staining (Fig. 10i) and stereological counts of TH^+^ and Nissl^+^ neurons (Fig. 10j,k) revealed a severe loss of nigral dopaminergic neurons (72% in MPTP-alone group and 74% in AAV-GFP/MPTP group compared with saline controls; *P*<0.001 for both groups) and total neurons (56% in MPTP-alone group and 55% in AAV-GFP/MPTP group compared with saline controls; *P*<0.001 for both groups) in both MPTP-alone and AAV-GFP/MPTP groups. In contrast, AAV-mediated delivery of PK2 significantly prevented the loss of nigral dopamine neurons and total neurons after MPTP treatment (Fig. 10j,k), with only 36% of dopamine neurons and 23% of total neurons degenerated compared with saline controls, further supporting the neuroprotective efficacy of PK2 overexpression (*P*<0.001 compared with MPTP-alone group; *P*<0.001 compared with AAV-GFP/MPTP group). The difference between TH^+^ and total neuronal loss is consistent with other reports from Przedborski and colleagues^38^ using the same dose (80 mg kg^−1^ of total MPTP) that we have used in an acute treatment paradigm, and could be due to the fact that TH protein levels can be transiently decreased after MPTP toxicity without directly causing neuronal death^39^. Combined with the above-mentioned observation that blocking PK2 signalling *in vivo* potentiates MPTP toxicity, these results convincingly demonstrate a protective role for PK2 signalling in the nigrostriatal dopaminergic system and suggest that modulating PK2 levels may be a novel therapeutic strategy for PD.

## Discussion

PD is a progressive neurodegenerative disorder with a complex multifactorial aetiology that makes therapeutic intervention a major challenge. Therapeutic approaches that can slow or halt disease progression are urgently needed. This study uncovers a novel neuroprotective role for the AVIT protein family member PK2 in nigral dopaminergic neurons with clinical relevance to PD pathophysiology. We demonstrate for the first time that PK2 is rapidly induced following neuronal injury in cell culture and preclinical mouse models of PD. We also confirmed elevated PK2 levels in nigral tissues of post-mortem PD patients, thereby establishing the clinical importance of these findings. Since the PKR2 receptor for PK2 is constitutively expressed on nigral dopaminergic neurons, pharmacological targeting of PK2 signalling could be explored for therapeutic development in PD. Our mechanistic studies demonstrate that both endogenous PK2 overexpression and soluble rPK2 protect dopaminergic neurons against mitochondrial dysfunction and cell death by promoting mitochondrial biogenesis through PGC-1α- and TFAM-dependent mechanisms, and by activating ERK and Akt signalling pathways. AAV-mediated PK2 gene delivery significantly protects mice against MPTP-induced behavioural deficits and dopaminergic degeneration in the nigrostriatal system, indicating that modulating PK2 activity may benefit the treatment of PD. We show that blocking the endogenous protective function of PK2 with the PK2 receptor antagonist PKRA7 worsens MPTP-induced histological, neurochemical and behavioural deficits, further demonstrating the neuroprotective function of PK2 in the dopaminergic system.

Our *in vivo* data from preclinical mouse models suggest that PK2 upregulation occurs during the early phase of dopaminergic degeneration before the onset of motor deficits. In the MPTP model, PK2 protein levels in nigral dopaminergic neurons increased 3 h post-MPTP administration and remained elevated for up to 3 days when nigral TH protein began declining (Fig. 2a). In the MitoPark transgenic mouse model of PD, PK2 increased by 8 weeks (Fig. 2i), before the onset of motor symptoms and remained elevated until 12 weeks when significant motor deficits are evident (Fig. 2g,h). Basal PK2 levels were either low or undetectable in the nigral brain region of healthy control mice. This is in agreement with previous reports showing little- or no-PK2 expression in regions of the midbrain^9^,^15^. Our finding that PK2 expression is upregulated early during the disease course before the onset of motor symptoms, together with our clinical data demonstrating that PK2 is elevated in PD patients, suggests that soluble circulating PK2 could serve as a potential marker of disease onset or progression. Understanding the transcriptional mechanisms underlying PK2 upregulation in dopaminergic neurons is crucial for targeting this pathway to achieve neuroprotection. Previous studies have shown that constitutive PK2 expression in the CNS is controlled by the circadian regulatory genes CLOCK/BMAL1 in the SCN and by Ngn1/MASH1 during olfactory bulb biogenesis^1^,^40^,^41^. The PK2 promoter is replete with multiple E-box sequences (CACGTG) that bind basic helix-loop-helix (bHLH) transcription factors such as HIF1α, which regulates PK2 expression during angiogenesis^12^. Others have reported HIF1α activation following MPTP-induced oxidative stress in nigral dopaminergic neurons and also in cell culture models of PD, making it a likely candidate for transcriptional upregulation of PK2 as a protective response^42^,^43^,^44^. Our laboratory is currently studying the transcriptional mechanisms regulating PK2 upregulation in dopaminergic neurons.

PK2 signalling supports the survival and proliferation of specific cell types outside the CNS^12^,^13^,^45^. For example, PK2 is required for angiogenesis and hematopoiesis, and regulates the survival of cardiomyocytes and myeloid cells via the AKT and STAT3 signalling pathways, respectively^46^,^47^. Also, MAPK/ERK activation is involved in PK2-mediated angiogenesis^23^,^24^. Biphasic PK2 expression has been demonstrated in the ischaemic striatum^48^. In agreement with these findings, we provide the first evidence that PK2 protects dopaminergic neuronal cells by activating ERK and Akt signalling pathways (Fig. 6). In addition, our results demonstrate for the first time that PK2 signalling via PKR2 on dopaminergic neurons upregulates neuroprotective antioxidant response pathways, particularly PGC-1α and TFAM (Fig. 7). Recent large-scale genome-wide association studies have shown that PGC-1α-responsive genes, which regulate cellular bioenergetics, are specifically downregulated in early PD patients^49^,^50^. Also, PGC-1α overexpression protects against dopaminergic degeneration in PD models^50^,^51^. The loss of functional TFAM signalling also results in dopaminergic degeneration and underlies the recently developed MitoPark genetic mouse model of PD. Our results suggest that PK2 is an upstream positive regulator of PGC-1α, and thus PK2 receptor agonists could have therapeutic potential in restoring mitochondrial dysfunction and mitigating progressive dopaminergic neuron loss in PD.

Overall, our results have important implications for understanding dopaminergic degeneration in PD and PK2 biology. To our knowledge, this is the first report showing that PK2 is induced early during dopaminergic neuronal injury in the adult brain and that recovery of PK2 levels translates into dopaminergic neuroprotection through activation of cell survival signalling events and restoration of mitochondrial function in preclinical models of PD. Thus, developing agonists for PK2 GPCRs could be a novel therapeutic strategy for slowing or arresting the progression of dopaminergic neuronal degeneration in PD.

## Methods

### Chemicals and reagents

DMEM/F-12, RPMI, neurobasal medium, B27 supplement, fetal bovine serum (FBS), L-glutamine, Lipofectamine 2000, G418, IR-dye tagged secondary antibodies, Hoechst nuclear stain, penicillin, streptomycin, MitoTracker Red dye and other cell culture reagents were purchased from Life Technologies (Gaithersburg, MD). Recombinant TNFα was purchased from Peprotech. Antibodies for rabbit (catalogue no. sc-67176) and goat (catalogue no. sc-48069) PK2, Bcl-2 (catalogue no. sc-7382), TFAM (catalogue no. sc-23588), TOM20 (catalogue no. sc-11021) and c-Myc (catalogue no. sc-40X) were purchased from Santa Cruz Biotechnology, Inc. (Santa Cruz, CA). Specificity of the PK2 rabbit antibody was confirmed by pre-absorption of antibody with rPK2 peptide (Supplementary Fig. 10). The PK2 hamster monoclonal antibody was obtained from Genentech (San Francisco, CA). Mouse TH (catalogue no. MAB318) and α-Tubulin (catalogue no. MAB1637) antibodies were purchased from Millipore (Temecula, CA). Prokineticin receptor R2 antibody (catalogue no. CVL-PAB0638-0) was obtained from Covalab (Villeurbanne, France). Antibodies for p44/42 (catalogue no. 9102), phospho-p44/42 (Thr202/Tyr204) (catalogue no. 4370, s), Akt (catalogue no. 4691, s) and phospho-Akt (Ser473) (catalogue no. 4060, s) were purchased from Cell Signaling (Danvers, MA), and goat polyclonal GFP antibody (catalogue no. ab6673) was obtained from Abcam (Cambridge, MA). The Bradford protein assay kit was purchased from Bio-Rad Laboratories (Hercules, CA). The Cell Titre Glo Luminescent Cell Viability assay kit was purchased from Promega. Caspase-3 assay substrate was purchased from MP Biomedicals (Solon, OH). The DNA fragmentation assay kit was purchased from Roche Applied Science. The PK2 antagonist PKRA7 (ref. 22) was kindly provided by Dr Qun-Yong Zhou (University of California, Irvine). The ROS generation determinant DCF-DA was purchased from Calbiochem (San Diego, CA). Mouse β-Actin antibody (catalogue no. A5441) and MPTP were obtained from Sigma (St Louis, MO).

### Animal studies

All animal procedures were approved by Iowa State University's Institutional Animal Care and Use Committee (IACUC). All mice were housed under a 12-h light cycle in a climate-controlled mouse facility (22±1 °C) with food and water available *ad libitum*. Based on the outcomes of preliminary studies, we estimated error variances of 15% for biochemical and neurochemical endpoints and 20% for histological measures. Setting *α*=0.05, we calculated sample sizes (*n*) of 8–10 animals per group, which has worked well for us in previous animal studies conducted in our laboratory^29^,^30^. We used all male animals for all studies. All mice were pre-screened for normal baseline performance during behavioural assessments conducted before randomly assigning animals to experimental groups. Investigators involved with data collection and analysis were not blinded to group allocation. MPTP studies were performed as previously described^52^, using C57BL/6NCrl mice (8–10 weeks of age) to determine levels of PK2 in the mouse brain. Over an 8 h period, one i.p. injection of 18 mg kg^−1^ MPTP or equal volumes of saline (vehicle) was administered every 2 h. Mice were euthanized at the indicated time points and samples processed for either IHC, qRT-PCR or western blotting. For PKRA7 experiments, C57BL/6 mice were injected i.p. with 18 mg kg^−1^ MPTP, or equal volumes of saline (vehicle), once daily for three consecutive days. PKRA7 (20 mg kg^−1^ per day) was given i.p. 24 h before MPTP treatment and continued once daily for 10 consecutive days until euthanization. For the PK2 rAAV neuroprotection experiments, C57BL/6NCrl mice were injected i.p. with 20 mg kg^−1^ MPTP, or equal volumes of saline (vehicle), once daily for four consecutive days and euthanized 24 h after the last MPTP injection.

### PK2-eGFP transgenic mice

The PK2-eGFP transgenic FVB/N-Swiss mice were originally generated at Rockefeller University by the GENSAT project using bacterial artificial chromosome (BAC) clone RP23-12A18, as described previously^19^. The eGFP reporter cassette included a polyadenylation site that prevents PK2 overexpression. The PK2-eGFP male mice (8–10 weeks of age) were obtained from NIH-supported Mutant Mouse Regional Resource Center, UC Davis, CA, and bred at Iowa State University's animal facility. Mice were housed under a 12-h light cycle in a climate-controlled mouse facility (22±1 °C) with food and water available *ad libitum*. Intraperitoneal injections of either 20 mg kg^−1^ MPTP or saline (vehicle) were administered every 2 h with a total of four injections.

### MitoPark transgenic mice

MitoPark transgenic mice were kindly provided by Dr Nils-Göran Larsson of Karolinska Institute, Stockholm, and were originally generated in his laboratory at the Max Planck Institute for Biology of Ageing by conditionally knocking out the mitochondrial transcription factor TFAM through control of the dopamine transporter promoter as previously described^20^,^21^. All mice used for this study were from the MitoPark breeding colony at Iowa State University. Housing was under a 12-h light cycle in a climate-controlled facility with food and water provided *ad libitum*. After behavioural experiments were performed at the indicated ages, mice were perfused for IHC or euthanized and dissected for western blot analysis. All animal procedures were approved by Iowa State University's Institutional Animal Care and Use Committee (IACUC).

### Human post-mortem PD brain samples

We obtained freshly frozen SN tissue samples and cryostat sections from the brains of confirmed post-mortem human PD patients and age-matched neurologically normal individuals from the brain banks at the Miller School of Medicine, University of Miami, FL, the Australian Brain Bank Network and the Banner Sun Health Research Institute, AZ. Case details of human post-mortem tissues are shown in Supplementary Table 2. For western blot experiments, tissue homogenates from freshly frozen tissue were prepared at a final concentration of 1 mg ml^−1^ total protein from which 40 μg of total protein was used. Nigral tissue blocks were fixed with 4% paraformaldehyde (PFA) solution (in 0.1 M phosphate-buffered saline, pH 7.4). Cryostat sections were used for IHC experiments. All human post-mortem samples were procured, stored and distributed according to the applicable regulations and guidelines involving consent, protection of human subjects and donor anonymity. For samples obtained from the Australian Brain Bank Network, Institutional Human Research Ethics Approval was obtained from the Medical Research Ethics Committee at the University of Queensland, Brisbane, Australia. Since the post-mortem human brain tissues were obtained from approved national brain banks, Institutional Review Board (IRB) approval from Iowa State University was not required.

### Fluorescent western blotting

Cells and micro-dissected brain tissues were collected at the end of treatment and lysed using a modified RIPA buffer as previously described^53^,^54^. Proteins were separated by sodium dodecyl sulfate (SDS) gel electrophoresis. Normalized protein samples were loaded into each well and first separated in a 4% stacking gel and then through a 12–18% resolving gel. Proteins were then transferred to a nitrocellulose membrane and blocked using fluorescent western blocking buffer (Rockland Immunochemicals). Membranes were washed several times in wash buffers comprising either PBS containing 0.05% Tween (PBST) or Tris-buffered saline containing 0.05% Tween (TBST). Primary antibodies, in either blocking buffer or 5% bovine serum albumin (BSA) solution, were then added to the membranes and incubated overnight at 4 °C. After another four to five washes, an infrared dye-tagged secondary antibody was added for 1 h. β-Actin or α-Tubulin was used as a loading control. Membranes were scanned using the Odyssey IR imaging system (LI-COR). Results shown in the figures are representative results and the uncropped original blot images are available in Supplementary Fig. 11.

### SYBR Green qRT-PCR

Total RNA was isolated from fresh cell pellets or animal tissues using the Absolutely RNA Miniprep kit from Agilent Technologies. First strand cDNA synthesis was performed using an Affinity Script qPCR cDNA synthesis system (Agilent Technologies). Real-time PCR was performed with the RT^2^ SYBR Green master mix (Qiagen) and pre-validated qPCR mouse primer sets were purchased from Qiagen. For normalization of each sample, the 18S rRNA gene (mouse and rat) was used as the housekeeping gene. The amount of each template was optimized empirically to maximize efficiency without inhibiting the PCR reaction. According to the manufacturer's guidelines, dissociation curves were run to ensure a single amplicon peak was obtained. The results are reported as fold change in gene expression, which was determined via the ΔΔC_t_ method using the threshold cycle (C_t_) value for the housekeeping gene and for the respective gene of interest in each sample.

### Mitochondrial fragmentation analysis

For mitochondrial dysfunction experiments, cells were plated onto glass-bottom dishes coated with 0.1% Poly-D-Lysine in RPMI with 10% FBS, penicillin (100 U ml^−1^), streptomycin (100 μg ml^−1^), and 2 mM L-glutamine. Cells were treated in RPMI with 2% FBS, penicillin (100 U ml^−1^), streptomycin (100 μg ml^−1^) and 2 mM-L-glutamine for 16 h. After treatment, MitoTracker Red dye was added to each dish at a dilution of 1:2,000 from a stock of 1 mM. The cells were incubated with the dye for 15–20 min at 37 °C. After incubation, cells were fixed with 4% PFA for 1 h and then washed several times with PBS. High-resolution confocal images were obtained using a Leica SP5 X confocal microscope system at the Confocal Microscopy and Image Analysis Facility at Iowa State University. Quantification of mitochondrial morphology was performed using a custom Image J macro program as described previously^55^,^56^. To determine the mitochondria/cell area ratio, mitochondrial area was measured by thresholding the MitoTracker fluorescence in ImageJ and dividing by the whole cell area.

### Mitochondrial DNA copy number

Total DNA containing both nuclear and mitochondrial DNA was isolated from N27 cells using the DNeasy Blood & Tissue kit (Qiagene). DNA concentrations were measured using Nanodrop ultraviolet absorbance spectrophotometry. Real-time PCR primers for mitochondrially encoded NADH dehydrogenase 1 (ND1) (forward, 5′-TGACCAACTAATGCACCTCCTA-3′; reverse, 5′-GAAAATTGGCAGGGAAATGT-3′) and nuclear-encoded glyceraldehyde phosphate dehydrogenase (GAPDH) (forward, 5′-AAACCCATCACCATCTTCCA-3′; reverse, 5′-CCTCGAAGTACCCTGTGCAT-3′) were synthesized as previously described^57^. For qPCR assay, each reaction contained 200 nM each of forward and reverse primers and 20 ng of sample DNA, plus appropriate amounts of Universal Master Mix (Qiagene). The standard cycling conditions were 95 °C for 5 min, followed by 40 cycles of 95 °C for 30 s and 60 °C for 30 s. The mtDNA levels were expressed as relative ND1 levels (ND1/GAPDH).

### CRISPR/Cas-based knockdown of PK2 in N27 cells

The lentivirus-based CRISPR/Cas9 KO plasmid, pLV-U6gRNA-Ef1aPuroCas9GFP-PK2, with the PK2 gRNA target sequence CTGTTCACACCGCCCGCCGGGG, was purchased from Sigma-Aldrich. To make lentivirus, the lenti-CRISPR/Cas9 PK2 KO plasmid and control plasmid were transfected into 293FT cells using the Mission Lentiviral Packaging Mix from Sigma-Aldrich according to the manufacturer's instructions. The lentivirus was harvested 48 h post transfection and titres were measured using the Lenti-X p24 Rapid Titer Kit (Clontech). For stable knockdown of PK2 in N27 cells, cells were grown in six-well plates with a seeding density of 0.1 × 10^6^ cells per well in growth media, and lentivirus was added to the media at an MOI of 100 the following morning. After 24 h, fresh media supplemented with puromycin (50 μg ml^−1^) was added to the cells for stable cell selection.

### Plasmid construction and rAAV generation

PK2 mRNA sequence was obtained from NCBI and codon-optimized for expression in the mouse using the GeneArt Gene Synthesis service at Life Technologies. The finished plasmid was sent to the Viral Vector Core Facility at the University of Iowa for cloning. Briefly, Kpn1 and Xho1 restriction enzymes were used to subclone the codon-optimized PK2 sequence into the pFBAAV2/5CMVmcsBgHpA viral vector. To generate rAAV, the pFBAAV2/5CMV-PK2-eGFP-BgHpA or AAV2/5CMVeGFP control viral plasmid was transfected into SF-9 insect cells along with the helper plasmid pAAV-RC containing Rep/Cap sequences for viral packaging. The resulting packaged cis-acting AAV baculovirus carries the AAV expression cassette flanked by the AAV inverted terminal repeats (ITR). P1 baculovirus stock was used to amplify the P2 virus. For production of the virus, SF-9 cells were grown in bioreactors and infected with the P2 virus. Cells were collected after signs of infection and collected in 50 ml tubes. Cells were pelleted and mechanically lysed with glass beads and a bead blender to release the virus. The lysate was treated with detergent to further lyse cells and dissociate virus from membranes and proteins. Benzonase was added to digest genomic, proviral and plasmid DNA while leaving packaged viral DNA intact. The lysate was then clarified by centrifugation to obtain viral particles. Viral particles were purified using Iodixanol step gradients of 25, 40 and 60%, and full viral particles were then collected at the 40–60% interface. The viral particles were further purified using a Mustang Q Anion Exchange disk as per manufacturer's instructions. A qPCR assay verified AAV2/5CMV-PK2-eGFP-BgHpa viral genome titre of 2.98 × 10^12^.

### Stereotaxic surgery

C57BL/6NCrl mice (8–10 weeks of age) were anaesthetized using a ketamine/xylazine mixture. The Angle 2 stereotaxic instrument was used with a 10-μl Hamilton syringe to inject the rAAV directly into the striatum at the following stereotaxic coordinates in relation to Bregma (mm): −2 ML, 0.5 AP, −4 DV. After drilling a hole in the skull, 3 μl of rAAV viral particles (∼9 × 10^12^ total viral particles) were injected into the brain. The animal was allowed to recover for 4 weeks to maximize viral gene expression before behavioural testing, euthanization or any treatment paradigm.

### Behavioural measurements

The automated VersaMax Monitor (model RXYZCM-16, AccuScan, Columbus, OH) was used to measure the spontaneous open-field locomotor activity of mice in an activity chamber made of clear Plexiglas and covered with a ventilated Plexiglas lid, as described previously^58^,^59^. Horizontal and vertical activity data were collected and analysed by a VersaMax Analyzer (model CDA-8, AccuScan). Locomotor activities were monitored during a 10-min test session following a 2-min acclimation period. For rotarod experiments, mice were placed on the rod as it rotated at a constant speed of 20 r.p.m. and latency to fall was recorded during 20-min sessions.

### HPLC analysis of striatal dopamine levels

HPLC samples were prepared and processed as described previously^30^. Briefly, mice were euthanized, striata were collected and neurotransmitters were extracted in 0.2 M perchloric acid solution containing 0.05% Na_2_EDTA, 0.1% Na_2_S_2_O_5_ and isoproterenol (internal standard). Dopamine and metabolites were separated isocratically by a reversed-phase column with a flow rate of 0.6 ml min^−1^ using a Dionex Ultimate 3000 HPLC system (pump ISO-3100SD, Thermo Scientific, Bannockburn, IL) equipped with a refrigerated automatic sampler (model WPS-3000TSL). The electrochemical detection system included a CoulArray model 5600A coupled with an analytical cell (microdialysis cell 5014B) and a guard cell (model 5020). Data acquisition and analysis were performed using Chromeleon 7 and ESA CoulArray 3.10 HPLC Software.

### Immunocytochemistry and histology

For immunocytochemistry, cells were plated onto coverslips in 24-well plates coated with 0.1% poly-D-lysine. After treatment, 4% formaldehyde was used to fix the cells for 30 min. The cells were washed four to five times with PBS. Blocking buffer containing 2% BSA, 0.1% Triton X-100 and 0.05% Tween 20 was added to the wells for 1 h to permeabilize the cells. The cells were incubated with primary PK2 (Rabbit polyclonal, 1:500 dilution), Bcl-2 (Mouse polyclonal, 1:500 dilution) or c-Myc (mouse monoclonal, 1:2,000) antibodies in 2% BSA at 4 °C overnight. After four to five washes, an Alexa dye-conjugated secondary antibody in 2% BSA was added and incubated at room temperature on a shaker for 1 h. Cells were then washed four to five times before adding Hoechst counterstain for 5 min to label nuclei. Coverslips were mounted onto slides with Fluoromount mounting media (Sigma). Cells were imaged under an Eclipse TE2000-U microscope (Nikon, Tokyo, Japan) with a SPOT digital camera (Diagnostic Instruments, Sterling Heights, MI), and all photomicrographs were processed in MetaMorph 5.7 (Universal Imaging, Downingtown, PA).

For histology, mice were perfused with saline and 4% PFA after being anaesthetized using a ketamine–xylazine mixture. Extracted brains were then post fixed in PFA for 24 h and cryoprotected in 30% sucrose before being embedded in OCT compound, frozen and sectioned on a Cryostat (CryoStar NX70, Thermo Scientific) at −20 °C. Antigen retrieval was performed using citrate buffer (10 mM sodium citrate, pH 8.5) at 90 °C for 30 min. Sections were then washed with PBS and permeabilized with blocking buffer (2% BSA, 0.1% Triton X-100 and 0.05% Tween 20 in PBS) for 1 h at room temperature. Antibodies directed against PK2 (rabbit polyclonal, 1:500), PKR1 (goat polyclonal, 1:1,000), PKR2 (rabbit polyclonal, 1:1,000) and TH (mouse monoclonal, 1:2,000) were incubated with the sections overnight at 4 °C. After several washes with PBS, sections were incubated with Alexa dye-conjugated secondary antibodies for 1 h at room temperature. Hoechst stain was added to the sections for 5 min at room temperature to label nuclei. Sections were then mounted on slides using the ProLong Gold antifade mounting medium (Molecular Probes) according to the manufacturer's instructions. A SPOT digital camera attached to an inverted fluorescence microscope (Nikon TE2000-U) was used to capture photomicrographs of sections.

DAB immunostaining was performed on striatal and SN sections, as described previously^30^,^60^. Briefly, 30-μm sections were incubated with either anti-TH antibody (Calbiochem, Billerica, MA, USA; rabbit anti-mouse, 1:1,800 dilution) or anti-PK2 followed by incubation with biotinylated anti-rabbit or goat or mouse secondary antibody. Separate sections were stained with cresyl violet to count the total number of Nissl-positive neurons in the same animals. Total numbers of TH^+^ and Nissl^+^ neurons in every sixth section of the SN were counted stereologically with Stereo Investigator software (MBF Bioscience, Williston, VT, USA) using an optical fractionator as described previously^61^.

Besides the above-mentioned methods, additional cell culture and assay methods have been described in detail in the Supplementary Methods section of the Supplementary Information, including methods for DAergic cell cultures, the generation of stable cell lines, and the following assays: Caspase-3 activity, DNA fragmentation, ATP production, intracellular ROS generation and Fluo-4 calcium mobilization.

### Data analysis and statistics

All *in vitro* data were determined from at least two biologically independent experiments, each done with a minimum of three biological replicates. Data analysis was performed using Prism 4.0 software (GraphPad Software, San Diego, CA). Data were analysed using one-way ANOVA and then Bonferroni's post-test comparison or two-way ANOVA to compare all treatment groups. Because our simple one-way and two-way ANOVAs were performed on completely balanced data sets, we ignored group variance inequalities that were all generated by large treatment effects. Where the normality assumption was violated we conducted nonparametric tests, however, in no case did the nonparametric results change the overall interpretation of our parametric results. Differences with *P*<0.05 were considered statistically significant. Student's *t*-test (two-tailed) was used when two groups were being compared.

### Data availability

All data are available from the authors upon request.

## Additional information

**How to cite this article**: Gordon, R. *et al*. Prokineticin-2 upregulation during neuronal injury mediates a compensatory protective response against dopaminergic neuronal degeneration. *Nat. Commun.* **7**, 12932 doi: 10.1038/ncomms12932 (2016).

## Supplementary Material

Supplementary Information Supplementary Figures 1-11, Supplementary Tables 1-2, Supplementary Methods and Supplementary References

## References

[b1] Cheng M. Y. . Prokineticin 2 transmits the behavioural circadian rhythm of the suprachiasmatic nucleus. Nature 417, 405–410 (2002). 12024206 10.1038/417405a

[b2] Hu W. P. . Impaired pain sensation in mice lacking prokineticin 2. Mol. Pain 2, 35 (2006). 17107623 10.1186/1744-8069-2-35 PMC1660571

[b3] Li J. D. . Attenuated circadian rhythms in mice lacking the prokineticin 2 gene. J. Neurosci. 26, 11615–11623 (2006). 17093083 10.1523/JNEUROSCI.3679-06.2006 PMC2713041

[b4] Lin D. C. . Identification and molecular characterization of two closely related G protein-coupled receptors activated by prokineticins/endocrine gland vascular endothelial growth factor. J. Biol. Chem. 277, 19276–19280 (2002). 11886876 10.1074/jbc.M202139200

[b5] Ng K. L. . Dependence of olfactory bulb neurogenesis on prokineticin 2 signaling. Science 308, 1923–1927 (2005). 15976302 10.1126/science.1112103

[b6] Pitteloud N. . Loss-of-function mutation in the prokineticin 2 gene causes Kallmann syndrome and normosmic idiopathic hypogonadotropic hypogonadism. Proc. Natl Acad. Sci. USA 104, 17447–17452 (2007). 17959774 10.1073/pnas.0707173104 PMC2077276

[b7] Cheng M. Y., Bittman E. L., Hattar S. & Zhou Q. Y. Regulation of prokineticin 2 expression by light and the circadian clock. BMC Neurosci. 6, 17 (2005). 15762991 10.1186/1471-2202-6-17 PMC555564

[b8] Zhou W., Li J. D., Hu W. P., Cheng M. Y. & Zhou Q. Y. Prokineticin 2 is involved in the thermoregulation and energy expenditure. Regul. Pept. 179, 84–90 (2012). 22960406 10.1016/j.regpep.2012.08.003

[b9] Cheng M. Y., Leslie F. M. & Zhou Q. Y. Expression of prokineticins and their receptors in the adult mouse brain. J. Comp. Neurol. 498, 796–809 (2006). 16927269 10.1002/cne.21087 PMC2667319

[b10] Gordon R., Anantharam V., Kanthasamy A. G. & Kanthasamy A. Proteolytic activation of proapoptotic kinase protein kinase Cdelta by tumor necrosis factor alpha death receptor signaling in dopaminergic neurons during neuroinflammation. J. Neuroinflammation 9, 82 (2012). 22540228 10.1186/1742-2094-9-82 PMC3419619

[b11] Soga T. . Molecular cloning and characterization of prokineticin receptors. Biochim. Biophys. Acta 1579, 173–179 (2002). 12427552 10.1016/s0167-4781(02)00546-8

[b12] LeCouter J. . The endocrine-gland-derived VEGF homologue Bv8 promotes angiogenesis in the testis: localization of Bv8 receptors to endothelial cells. Proc. Natl Acad. Sci. USA 100, 2685–2690 (2003). 12604792 10.1073/pnas.0337667100 PMC151401

[b13] Shojaei F. . Bv8 regulates myeloid-cell-dependent tumour angiogenesis. Nature 450, 825–831 (2007). 18064003 10.1038/nature06348

[b14] Li M., Bullock C. M., Knauer D. J., Ehlert F. J. & Zhou Q. Y. Identification of two prokineticin cDNAs: recombinant proteins potently contract gastrointestinal smooth muscle. Mol. Pharmacol. 59, 692–698 (2001). 11259612 10.1124/mol.59.4.692

[b15] Zhang C., Truong K. K. & Zhou Q. Y. Efferent projections of prokineticin 2 expressing neurons in the mouse suprachiasmatic nucleus. PloS ONE 4, e7151 (2009). 19784373 10.1371/journal.pone.0007151 PMC2747004

[b16] Cheng M. Y., Leslie F. M. & Zhou Q. Y. Expression of prokineticins and their receptors in the adult mouse brain. J. Comp. Neurol. 498, 796–809 (2006). 16927269 10.1002/cne.21087 PMC2667319

[b17] Masuda Y. . Isolation and identification of EG-VEGF/prokineticins as cognate ligands for two orphan G-protein-coupled receptors. Biochem. Biophys. Res. Commun. 293, 396–402 (2002). 12054613 10.1016/S0006-291X(02)00239-5

[b18] Heintz N. Gene expression nervous system atlas (GENSAT). Nat. Neurosci. 7, 483 (2004). 15114362 10.1038/nn0504-483

[b19] Gong S. . A gene expression atlas of the central nervous system based on bacterial artificial chromosomes. Nature 425, 917–925 (2003). 14586460 10.1038/nature02033

[b20] Ekstrand M. I. & Galter D. The MitoPark mouse - an animal model of Parkinson's disease with impaired respiratory chain function in dopamine neurons. Parkinsonism Relat. Disord. 15, (Suppl 3): S185–S188 (2009). 20082987 10.1016/S1353-8020(09)70811-9

[b21] Ekstrand M. I. . Progressive parkinsonism in mice with respiratory-chain-deficient dopamine neurons. Proc. Natl Acad. Sci. USA 104, 1325–1330 (2007). 17227870 10.1073/pnas.0605208103 PMC1783140

[b22] Curtis V. F. . A PK2/Bv8/PROK2 antagonist suppresses tumorigenic processes by inhibiting angiogenesis in glioma and blocking myeloid cell infiltration in pancreatic cancer. PloS ONE 8, e54916 (2013). 23372791 10.1371/journal.pone.0054916 PMC3553000

[b23] Guilini C. . Divergent roles of prokineticin receptors in the endothelial cells: angiogenesis and fenestration. Am. J. Physiol. Heart Circ. Physiol. 298, H844–H852 (2010). 20023120 10.1152/ajpheart.00898.2009

[b24] Lin D. C. . Identification and molecular characterization of two closely related G protein-coupled receptors activated by prokineticins/endocrine gland vascular endothelial growth factor. J. Biol. Chem. 277, 19276–19280 (2002). 11886876 10.1074/jbc.M202139200

[b25] Ngan E. S. & Tam P. K. Prokineticin-signaling pathway. Int. J. Biochem. Cell Biol. 40, 1679–1684 (2008). 18440852 10.1016/j.biocel.2008.03.010

[b26] Martin C. . The role of the prokineticin 2 pathway in human reproduction: evidence from the study of human and murine gene mutations. Endocr. Rev. 32, 225–246 (2011). 21037178 10.1210/er.2010-0007 PMC3365793

[b27] Crews C. M., Alessandrini A. & Erikson R. L. The primary structure of MEK, a protein kinase that phosphorylates the ERK gene product. Science 258, 478–480 (1992). 1411546 10.1126/science.1411546

[b28] Kim D. . A small molecule inhibits Akt through direct binding to Akt and preventing Akt membrane translocation. J. Biol. Chem. 285, 8383–8394 (2010). 20068047 10.1074/jbc.M109.094060 PMC2832988

[b29] Ghosh A. . Anti-inflammatory and neuroprotective effects of an orally active apocynin derivative in pre-clinical models of Parkinson's disease. J. Neuroinflammation 9, 241 (2012). 23092448 10.1186/1742-2094-9-241 PMC3488558

[b30] Ghosh A. . The peptidyl-prolyl isomerase Pin1 up-regulation and proapoptotic function in dopaminergic neurons: relevance to the pathogenesis of Parkinson disease. J. Biol. Chem. 288, 21955–21971 (2013). 23754278 10.1074/jbc.M112.444224 PMC3724650

[b31] Thomas B. . Resistance to MPTP-neurotoxicity in alpha-synuclein knockout mice is complemented by human alpha-synuclein and associated with increased beta-synuclein and Akt activation. PloS ONE 6, e16706 (2011). 21304957 10.1371/journal.pone.0016706 PMC3031616

[b32] De Miranda B. R. . Novel para-phenyl substituted diindolylmethanes protect against MPTP neurotoxicity and suppress glial activation in a mouse model of Parkinson's disease. Toxicol. Sci. 143, 360–373 (2015). 25406165 10.1093/toxsci/kfu236 PMC4306720

[b33] St-Amour I. . Impact of intravenous immunoglobulin on the dopaminergic system and immune response in the acute MPTP mouse model of Parkinson's disease. J. Neuroinflammation 9, 234 (2012). 23046563 10.1186/1742-2094-9-234 PMC3520736

[b34] Kirik D., Rosenblad C., Bjorklund A. & Mandel R. J. Long-term rAAV-mediated gene transfer of GDNF in the rat Parkinson's model: intrastriatal but not intranigral transduction promotes functional regeneration in the lesioned nigrostriatal system. J. Neurosci. 20, 4686–4700 (2000). 10844038 10.1523/JNEUROSCI.20-12-04686.2000 PMC6772474

[b35] Klein R. L., Lewis M. H., Muzyczka N. & Meyer E. M. Prevention of 6-hydroxydopamine-induced rotational behavior by BDNF somatic gene transfer. Brain Res. 847, 314–320 (1999). 10575102 10.1016/s0006-8993(99)02116-2

[b36] Chen X. . The sirtuin-2 inhibitor AK7 is neuroprotective in models of Parkinson's disease but not amyotrophic lateral sclerosis and cerebral ischemia. PloS ONE 10, e0116919 (2015). 25608039 10.1371/journal.pone.0116919 PMC4301865

[b37] Carta A. R. . Inactivation of neuronal forebrain A receptors protects dopaminergic neurons in a mouse model of Parkinson's disease. J. Neurochem. 111, 1478–1489 (2009). 19817968 10.1111/j.1471-4159.2009.06425.x PMC2820161

[b38] Jackson-Lewis V., Jakowec M., Burke R. E. & Przedborski S. Time course and morphology of dopaminergic neuronal death caused by the neurotoxin 1-methyl-4-phenyl-1,2,3,6-tetrahydropyridine. Neurodegeneration 4, 257–269 (1995). 8581558 10.1016/1055-8330(95)90015-2

[b39] Meredith G. E. & Rademacher D. J. MPTP mouse models of Parkinson's disease: an update. J. Parkinsons Dis. 1, 19–33 (2011). 23275799 10.3233/JPD-2011-11023 PMC3530193

[b40] Cheng M. Y., Bittman E. L., Hattar S. & Zhou Q. Y. Regulation of prokineticin 2 expression by light and the circadian clock. BMC Neurosci. 6, 17 (2005). 15762991 10.1186/1471-2202-6-17 PMC555564

[b41] Zhang C. . Prokineticin 2 is a target gene of proneural basic helix-loop-helix factors for olfactory bulb neurogenesis. J. Biol. Chem. 282, 6917–6921 (2007). 17259180 10.1074/jbc.C600290200

[b42] Lee D. W. . Inhibition of prolyl hydroxylase protects against 1-methyl-4-phenyl-1,2,3,6-tetrahydropyridine-induced neurotoxicity: model for the potential involvement of the hypoxia-inducible factor pathway in Parkinson disease. J. Biol. Chem. 284, 29065–29076 (2009). 19679656 10.1074/jbc.M109.000638 PMC2781452

[b43] Correia S. C. & Moreira P. I. Hypoxia-inducible factor 1: a new hope to counteract neurodegeneration? J. Neurochem. 112, 1–12 (2010). 19845827 10.1111/j.1471-4159.2009.06443.x

[b44] Sharp F. R. & Bernaudin M. HIF1 and oxygen sensing in the brain. Nat. Rev. Neurosci. 5, 437–448 (2004). 15152194 10.1038/nrn1408

[b45] LeCouter J., Zlot C., Tejada M., Peale F. & Ferrara N. Bv8 and endocrine gland-derived vascular endothelial growth factor stimulate hematopoiesis and hematopoietic cell mobilization. Proc. Natl Acad. Sci. USA 101, 16813–16818 (2004). 15548611 10.1073/pnas.0407697101 PMC528996

[b46] Urayama K. . The prokineticin receptor-1 (GPR73) promotes cardiomyocyte survival and angiogenesis. Faseb J. 21, 2980–2993 (2007). 17442730 10.1096/fj.07-8116com

[b47] Xin H. . G-protein-coupled receptor agonist BV8/prokineticin-2 and STAT3 protein form a feed-forward loop in both normal and malignant myeloid cells. J. Biol. Chem. 288, 13842–13849 (2013). 23548897 10.1074/jbc.M113.450049 PMC3650420

[b48] Cheng M. Y. . Prokineticin 2 is an endangering mediator of cerebral ischemic injury. Proc. Natl Acad. Sci. USA 109, 5475–5480 (2012). 22431614 10.1073/pnas.1113363109 PMC3325724

[b49] Zheng B. . PGC-1alpha, a potential therapeutic target for early intervention in Parkinson's disease. Sci. Transl. Med. 2, 52ra73 (2010). 10.1126/scitranslmed.3001059 PMC3129986 20926834

[b50] Mudo G. . Transgenic expression and activation of PGC-1alpha protect dopaminergic neurons in the MPTP mouse model of Parkinson's disease. Cell. Mol. Life Sci. 69, 1153–1165 (2012). 21984601 10.1007/s00018-011-0850-z PMC11114858

[b51] O'Donnell K. C. . Axon degeneration and PGC-1alpha-mediated protection in a zebrafish model of alpha-synuclein toxicity. Dis. Model. Mech. 7, 571–582 (2014). 24626988 10.1242/dmm.013185 PMC4007408

[b52] Zhang D., Anantharam V., Kanthasamy A. & Kanthasamy A. G. Neuroprotective effect of protein kinase C delta inhibitor rottlerin in cell culture and animal models of Parkinson's disease. J. Pharmacol. Exp. Ther. 322, 913–922 (2007). 17565007 10.1124/jpet.107.124669

[b53] Kanthasamy A. G. . A novel peptide inhibitor targeted to caspase-3 cleavage site of a proapoptotic kinase protein kinase C delta (PKCdelta) protects against dopaminergic neuronal degeneration in Parkinson's disease models. Free Radic. Biol. Med. 41, 1578–1589 (2006). 17045926 10.1016/j.freeradbiomed.2006.08.016

[b54] Latchoumycandane C., Anantharam V., Jin H., Kanthasamy A. & Kanthasamy A. Dopaminergic neurotoxicant 6-OHDA induces oxidative damage through proteolytic activation of PKCdelta in cell culture and animal models of Parkinson's disease. Toxicol. Appl. Pharmacol. 256, 314–323 (2011). 21846476 10.1016/j.taap.2011.07.021 PMC3205342

[b55] Dagda R. K. . Loss of PINK1 function promotes mitophagy through effects on oxidative stress and mitochondrial fission. J. Biol. Chem. 284, 13843–13855 (2009). 19279012 10.1074/jbc.M808515200 PMC2679485

[b56] Merrill R. A. . Mechanism of neuroprotective mitochondrial remodeling by PKA/AKAP1. PLoS Biol. 9, e1000612 (2011). 21526220 10.1371/journal.pbio.1000612 PMC3079583

[b57] McInerny S. C., Brown A. L. & Smith D. W. Region-specific changes in mitochondrial D-loop in aged rat CNS. Mech. Ageing Dev. 130, 343–349 (2009). 19428453 10.1016/j.mad.2009.01.008

[b58] Ngwa H. A., Kanthasamy A., Jin H., Anantharam V. & Kanthasamy A. G. Vanadium exposure induces olfactory dysfunction in an animal model of metal neurotoxicity. Neurotoxicology 43, 73–81 (2013). 24362016 10.1016/j.neuro.2013.12.004 PMC4062607

[b59] Harischandra D. S. . Role of proteolytic activation of protein kinase Cdelta in the pathogenesis of prion disease. Prion 8, 143–153 (2014). 24576946 10.4161/pri.28369 PMC4988799

[b60] Roy A. . Sodium phenylbutyrate controls neuroinflammatory and antioxidant activities and protects dopaminergic neurons in mouse models of Parkinson's disease. PloS ONE 7, e38113 (2012). 22723850 10.1371/journal.pone.0038113 PMC3377667

[b61] Ghosh A. . Neuroprotection by a mitochondria-targeted drug in a Parkinson's disease model. Free Radic. Biol. Med. 49, 1674–1684 (2010). 20828611 10.1016/j.freeradbiomed.2010.08.028 PMC4020411

