# Supplementary material for: Prokineticin-2 upregulation during neuronal injury mediates a compensatory protective response against dopaminergic neuronal degeneration
Source: Nat Commun. 2016 Oct 5;7:12932. doi: 10.1038/ncomms12932 (PMC5059486; doi:10.1038/ncomms12932)
Supplement: Supplementary Information — Supplementary Figures 1-11, Supplementary Tables 1-2, Supplementary Methods and Supplementary References [file ncomms12932-s1.pdf]

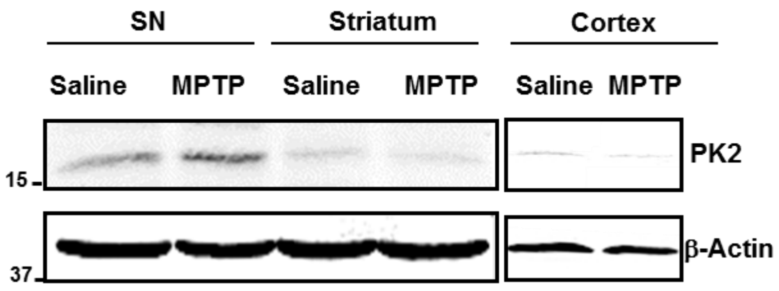

Supplementary Figure 1. Prokineticin-2 is induced in the substantia nigra, but not in the striatum, in MPTP-treated mice. C57BL/6 mice were injected with 4 doses of MPTP (18 mg/kg, i.p.) each separated by 2-h intervals. Representative Western blots of PK2 expression in nigral, striatal and cortical lysates.

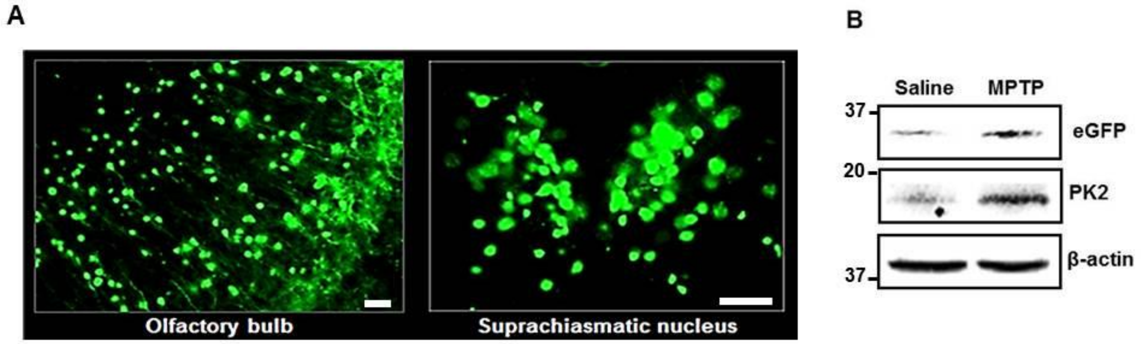

Supplementary Figure 2. (*A*) Direct visualization of eGFP-PK2 in the olfactory bulb and suprachiasmatic nucleus where PK2 is known to be constitutively expressed at high levels in the adult PK2-eGFP reporter transgenic mouse brain. Representative images are shown. Scale bars, 40 µm. (*B*) Saline- or MPTP-treated transgenic mice were sacrificed 24 h after the last injection and subjected to immunoblotting analysis of eGFP and PK2 expression in substantia nigra; β-Actin was used as a loading control.

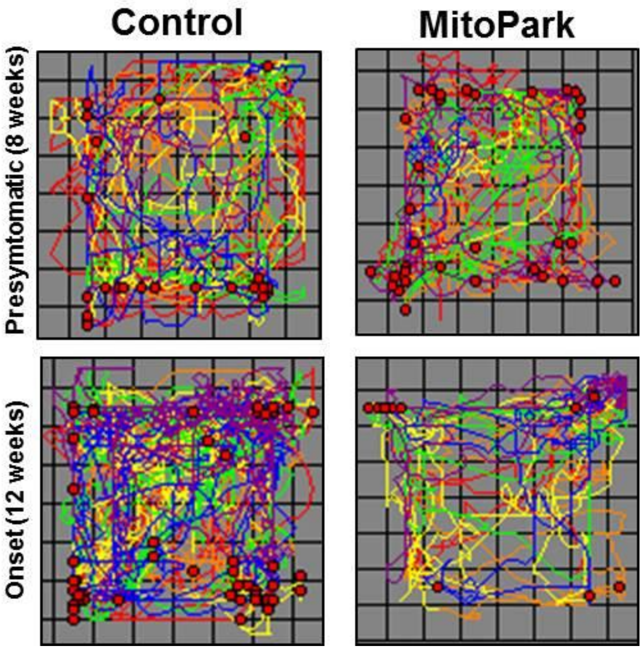

Supplementary Figure 3. Spontaneous locomotor activity plots of MitoPark mice and age-matched controls at 8 weeks (presymptomatic) and 12 weeks (onset) showing decreased activity in MitoPark mice with disease onset at 12 weeks.

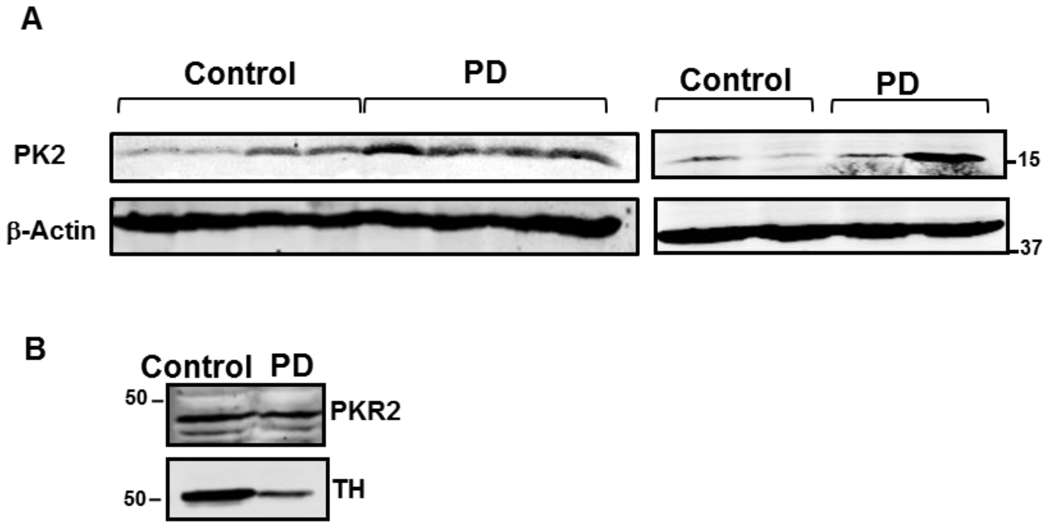

Supplementary Figure 4. Additional Western blots of PK2 **(A)** and PKR2 **(B)** expression in nigral lysates PD patients and age-matched controls.

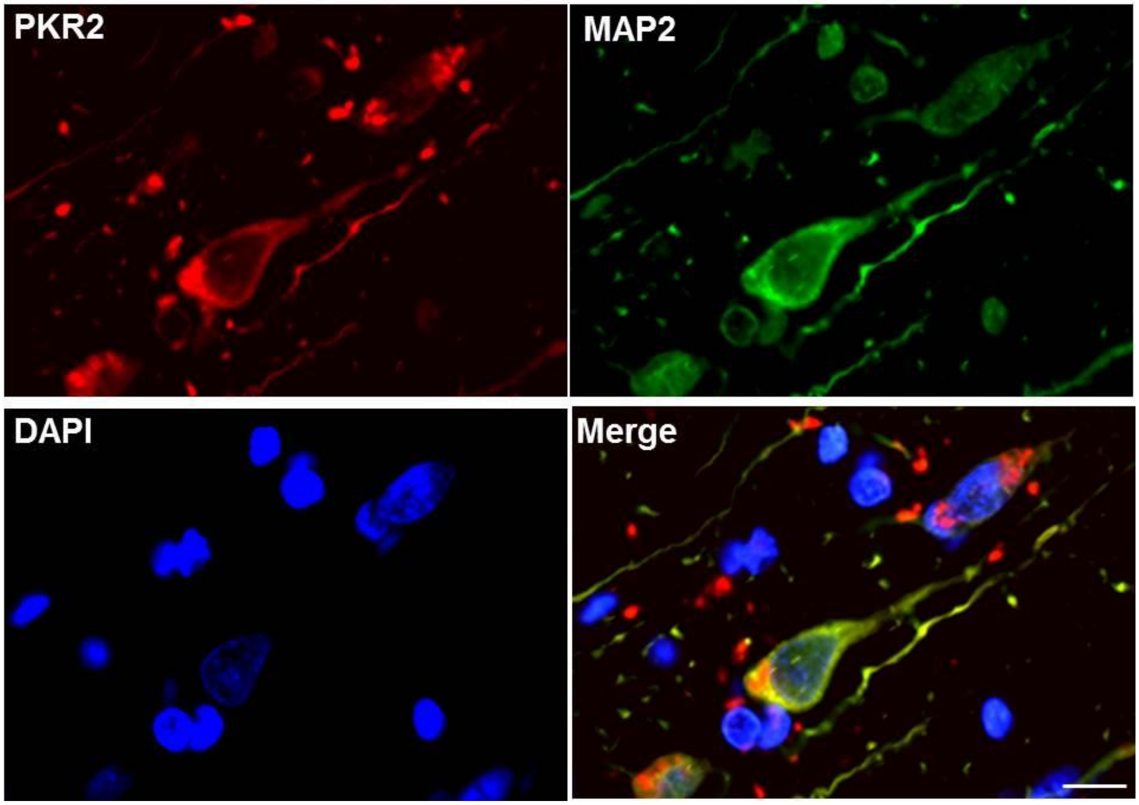

Supplementary Figure 5. PKR2 is expressed ubiquitously in MAP2-positive neurons in the substantia nigra of human brains. Double-labeling immunofluorescence for PKR2 (Red), MAP2 (Green) and nuclei (blue) in substantial nigra of human brains. Scale bar, 10 µm. Representative images are shown.

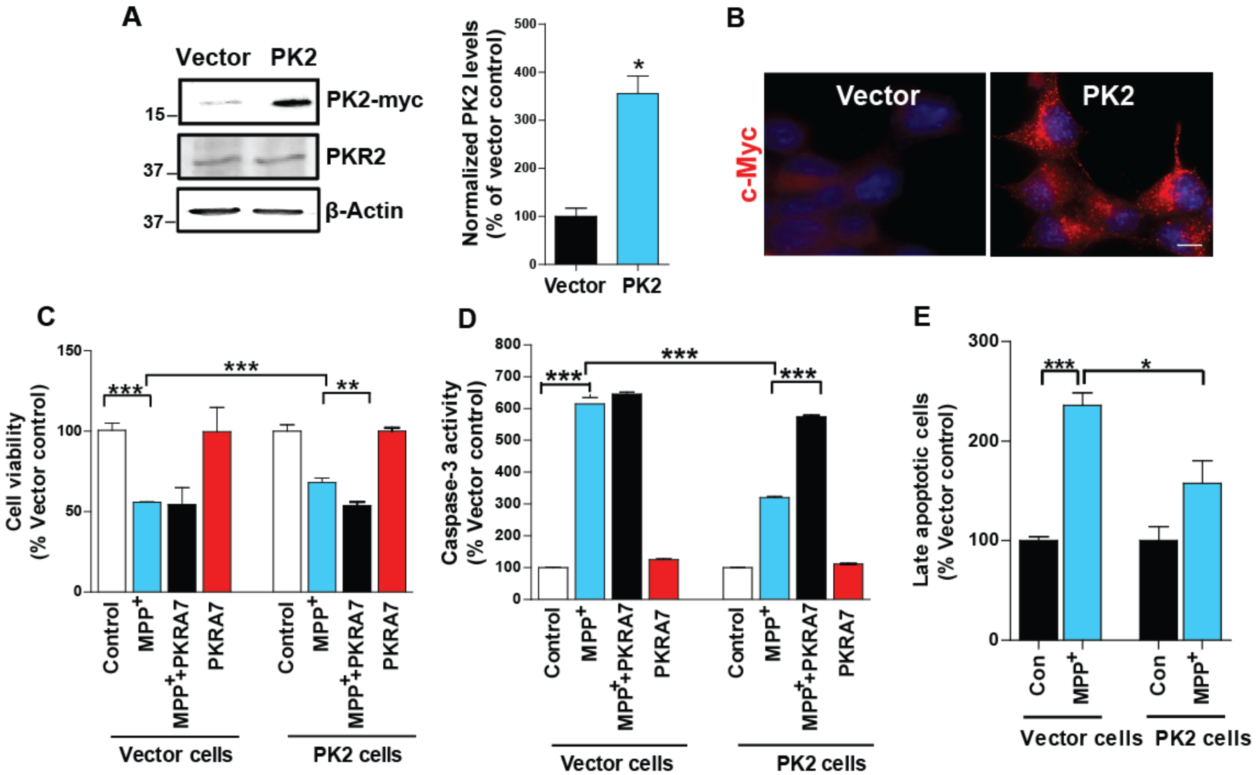

Supplementary Figure 6. *Stable expression of PK2 protects against dopaminergic cell death.* **(A)** Stable expression of PK2 in dopaminergic MN9D cells was confirmed by Western blot. Overexpression of PK2 did not affect PK2 receptor expression. Densitometric analysis of PK2 band intensity is shown in right panel with a total n = 2 and analyzed using student's t-test. (**B**) Immunofluorescence staining for PK2-myc expression in vector and PK2 stable cells using an myc-antibody. Scale bar, 10μm. (**C-D**) MN9D cells expressing PK2 were partially protected from MPP^+-induced neurotoxic insult as assessed by MTS (C) (student's t-test; n = 3-5) and caspase-3 activity assays (D) (one-way ANOVA with Bonferroni post-test; n = 3). The protective effect of PK2 overexpression was abolished using the specific PK2 receptor antagonist PKRA7 (2 μM). (**E**) The number of late-apoptotic cells following MPP^+ treatment was abolished in PK2-overexpressing cells at 24 h, as quantified by flow cytometric analysis of Annexin V staining (one-way ANOVA with Bonferroni post-test; n = 3). Data represented by group mean ± SEM and asterisks denote statistical significance (* p<0.05 and *** p<0.001, relative to relevant control or MPP^+-treatment groups).

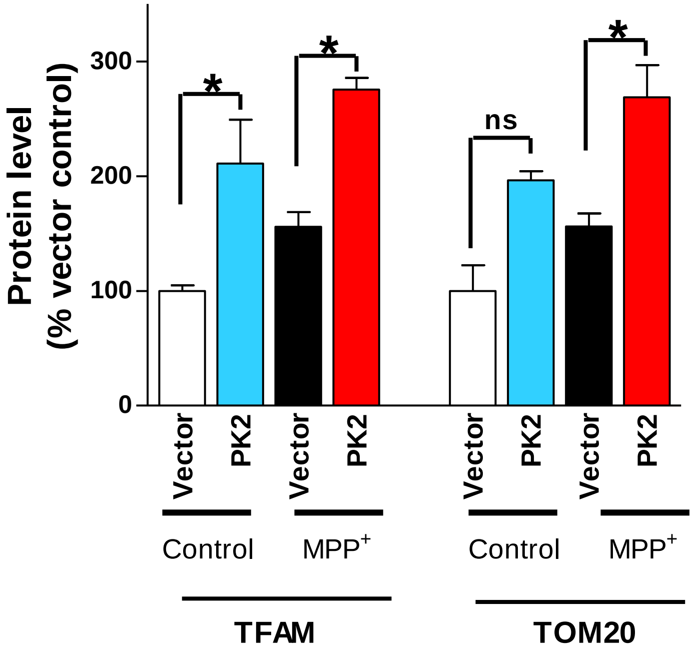

Supplementary Figure 7. Densitometric analysis of the Western blots in Figure 7J. Data are representative of at least three independent experiments (Means ± SEMs of three samples) (* $p<0.05$, relative to Vector control or MPP^+-treatment groups, ns, non-significant $p = 0.1618$).

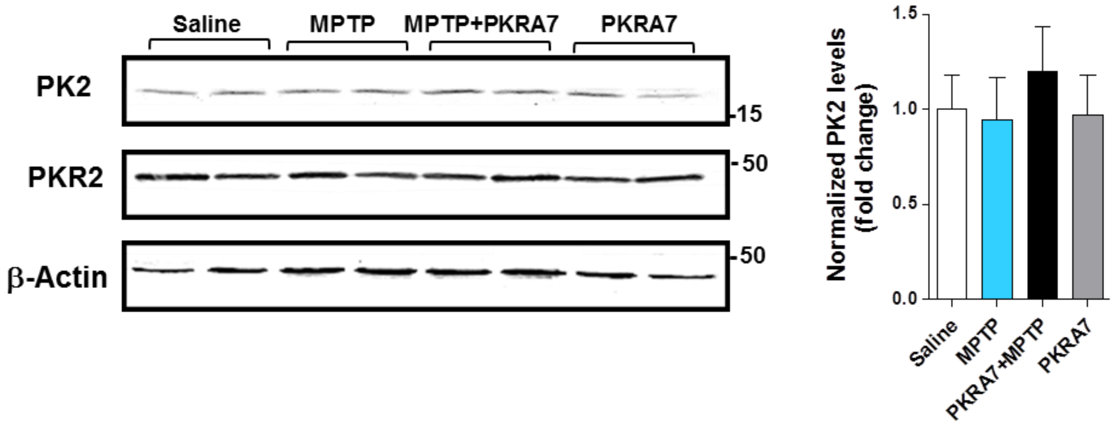

Supplementary Figure 8. Mice were intraperitoneally injected with 18 mg/kg MPTP or equal volumes of saline (vehicle), once daily for three consecutive days. PKRA7 (20 mg/kg/day) was given i.p. 24 h before MPTP treatment and continued once daily for 10 consecutive days until sacrifice. Western blot analysis of SN samples demonstrates that PKRA7 treatment does not change PK2 or PKR2 levels. Data represented by group mean ± SEM using one-way ANOVA with Bonferroni post-test and 4 mice per group.

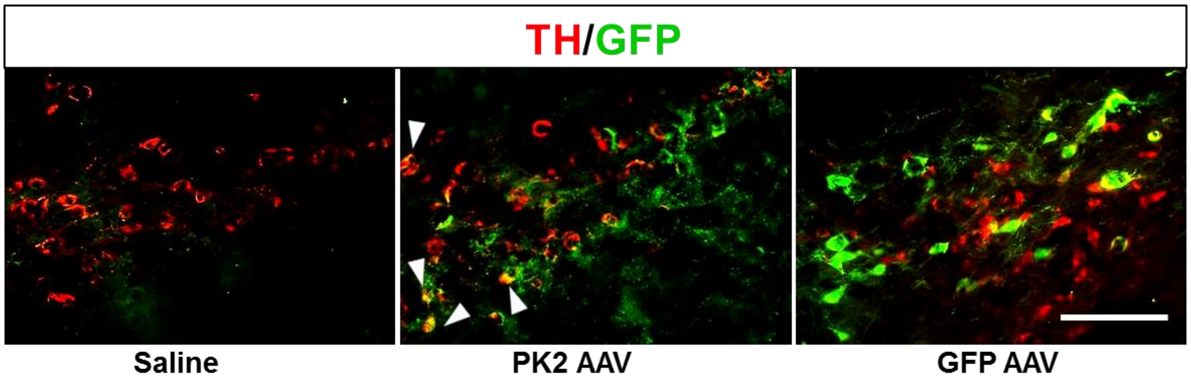

Supplementary Figure 9. Expression of GFP-PK2 in the substantia nigra following PK2 AAV injection into the striatum at 4 weeks post injection (white arrows). Double labeling immunofluorescence for GFP (Green) and TH (Red) in substantia nigra region of mouse brains stereotaxically injected with Saline, PK2 AAV, or GFP AAV in the striatum. Scale bar, 40 µm.

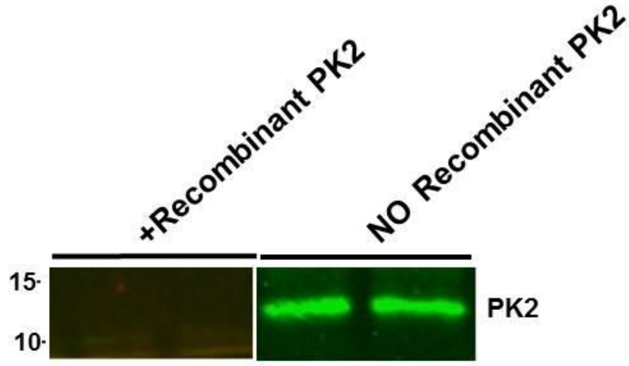

Supplementary Figure 10. Rabbit PK2 polyclonal antibody was pre-absorbed by incubating 5 µg of antibody together with 25 µg of recombinant PK2 protein overnight and Western blot was run with N27 cell lysates and incubated overnight with either pre-absorbed antibody (left) or normal antibody (right).

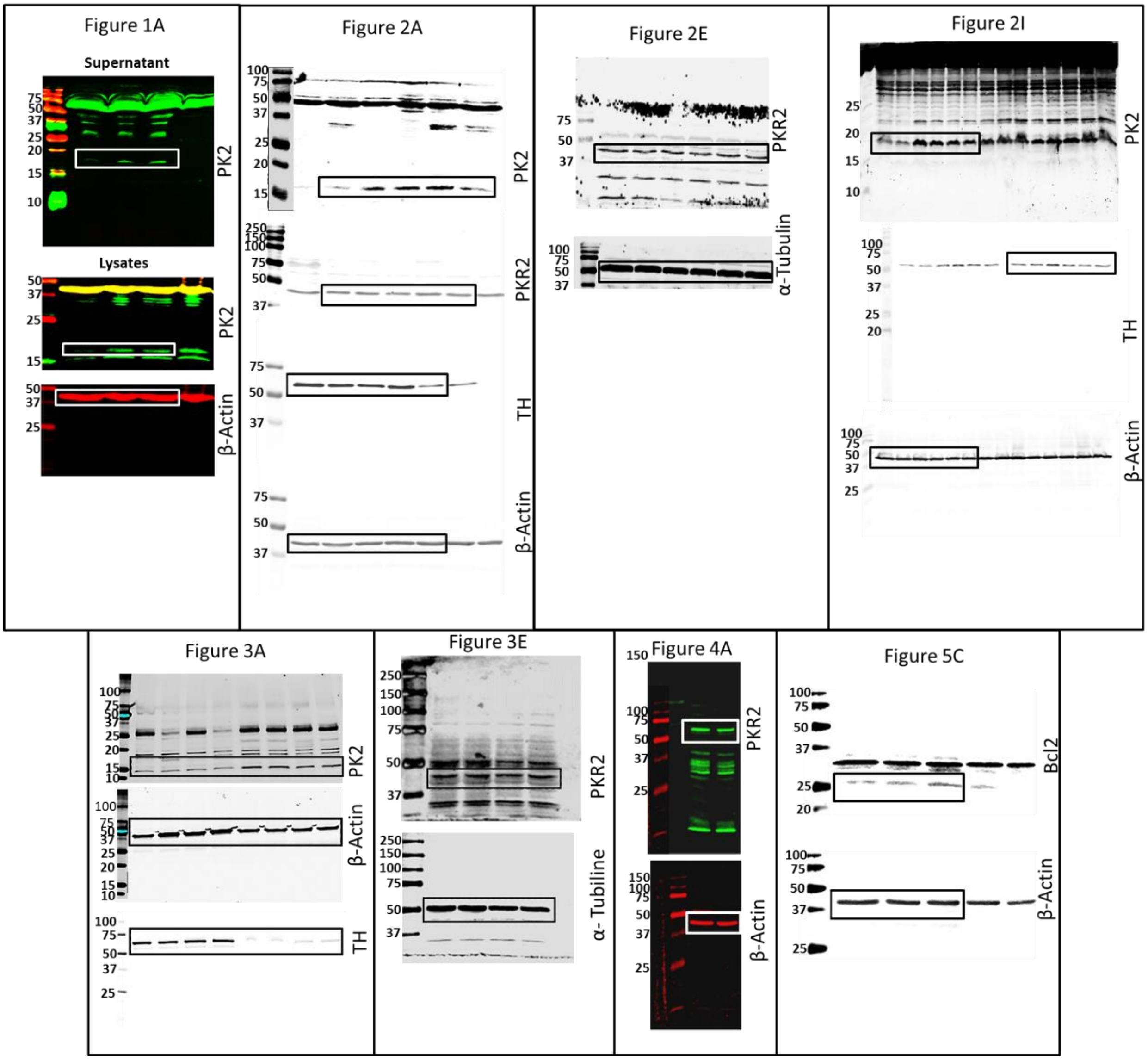

Figure 1A
Supernatant
PK2
Lysates
PK2
β-Actin
Figure 2A
PK2
PKR2
TH
β-Actin
Figure 2E
PKR2
α-Tubulin
Figure 2I
PK2
TH
β-Actin
Figure 3A
PK2
β-Actin
TH
Figure 3E
PKR2
α- Tubiline
Figure 4A
PKR2
β-Actin
Figure 5C
Bcl2
β-Actin

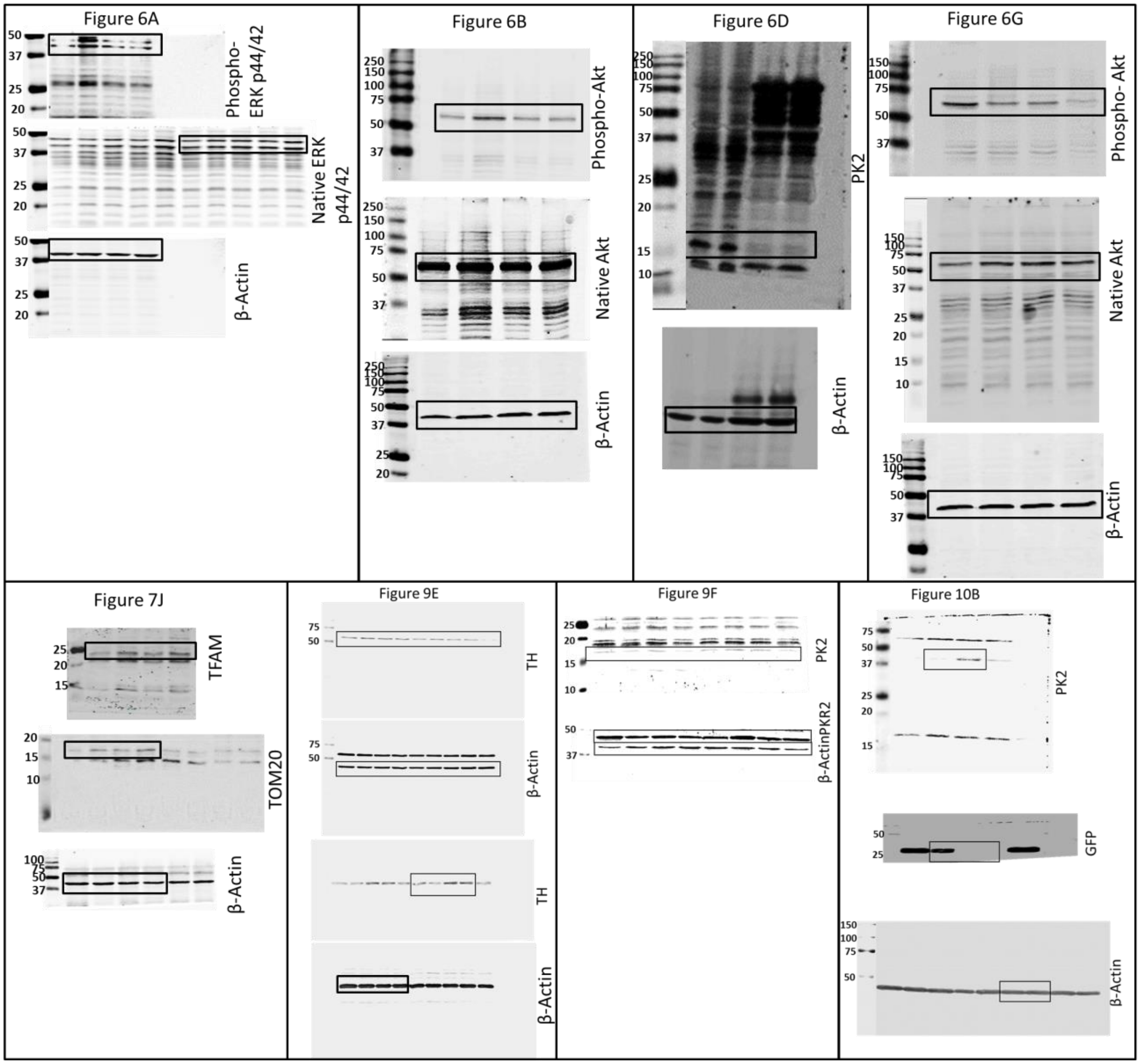

Figure 6A
Phospho-ERK p44/42
Native ERK p44/42
β-Actin
Figure 6B
Phospho-Akt
Native Akt
β-Actin
Figure 6D
PK2
β-Actin
Figure 6G
Phospho- Akt
Native Akt
β-Actin
Figure 7J
TFAM
TOM20
β-Actin
Figure 9E
TH
β-Actin
TH
β-Actin
Figure 9F
PK2
β-ActinPKR2
Figure 10B
PK2
GFP
β-Actin

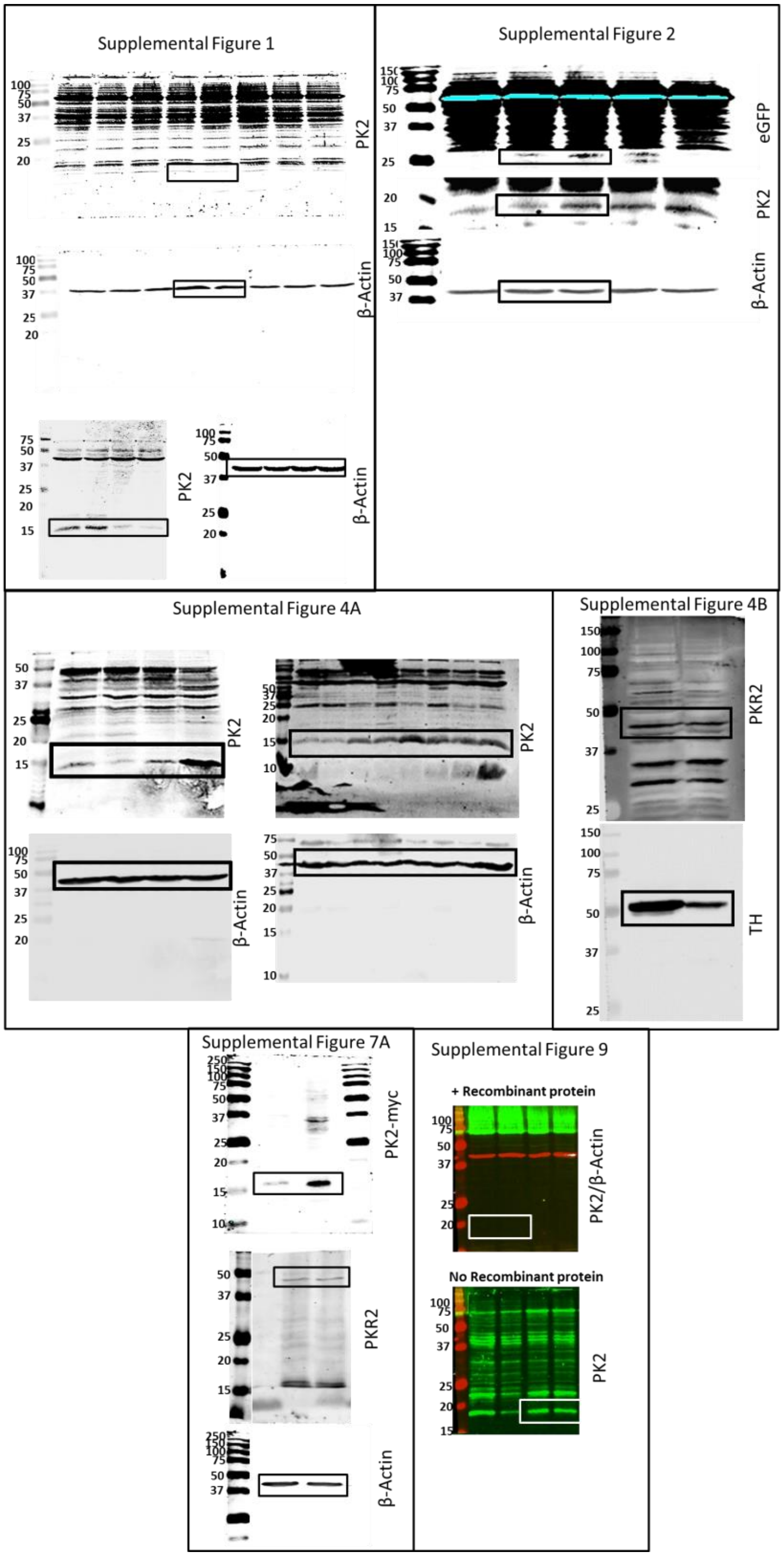

Supplementary Figure 11. Full images of blots shown in the main text and supplementary figures.

Supplementary Table 1. TNFα-induced upregulation of PK2 gene expression in N27 dopaminergic cells as assessed via qPCR array

Gene	Fold change (TNFα/Control)
Prok2	6.81
Ripk2	3.80
Tnfrsf5	2.86

PK2 was discovered as a candidate in a Qiagen mouse apoptosis qPCR array study of 84 apoptosis-related genes. Following TNFα exposure, the three most upregulated genes are shown with PK2 mRNA expression induced by over 6-fold.

Supplementary Table 2. Human post-mortem tissues used for immunoblot and IHC in Fig. 3 and supplementary Fig. 4. (N/A= not available)

**Sample Code**	**Gender**	**Age (yrs)**	**Post-mortem intervals**	**Braak Score**	**Neuropathological Diagnosis**
**454**	M	69	40	I-II	Parkinson's disease
**580**	M	65	27	III-IV	Parkinson's disease
**588**	M	72	12	III-IV	Parkinson's disease
**582**	F	76	36	N/A	Parkinson's disease
**526**	F	54	23		Normal control
**540**	M	64	28		Normal control
**562**	M	42	13		Normal control
**560**	F	56	5.42		Normal control

**Code**	**Gender**	**Age (yrs)**	**Race**	**Post-mortem intervals**	**Braak Score**	**Neuropathological Diagnosis**
**AK-2013-1**	M	83	C	10.5	N/A	Parkinson's disease
**AK-2013-2**	F	88	C	3.8	N/A	Parkinson's disease
**AK-2013-3**	M	81	C	6	N/A	Parkinson's disease
**AK-12-03**	F	87	C	2.08	IV	Parkinson's disease
**AK-11-54**	F	76	C	3.5	III	Parkinson's disease
**AK-11-90**	M	78	C	2.75	III	Parkinson's disease
**AK-2013-4**	F	87	C	12		Normal control
**AK-2013-5**	F	72	C	7.5		Normal control
**AK-2013-6**	F	87	C	8		Normal control
**AK-00-14**	M	90	C	4		Normal control
**AK-05-12**	M	88	C	2		Normal control
**AK-11-11**	M	93	C	2.15		Normal control

Supplementary Methods

DAergic cell cultures and generation of stable cell lines

A rat mesencephalic dopaminergic clonal neural cell model (N27 neuronal cells) was a kind gift from Dr. Kedar N. Prasad (University of Colorado Health Sciences Center, Denver, CO) and grown in RPMI 1640 media supplemented with 10% FBS, penicillin (100 U/ml), streptomycin (100 μg/ml), and 2 mM L-glutamine in 175-mm^3 flasks, as previously described [1, 2, 3]. N27 cells were maintained in an incubator at 37°C with 5% CO_2. To generate N27 cell lines stably overexpressing human PK2, N27 cells were stably infected with lentiviral particles encoding human PK2-GFP or GFP-alone. For the production of lentiviruses, lentiviral constructs (pLenti-PK2-mGFP and pLenti-C-mGFP, Origene, Rockville, MD) were packaged into viruses via transient transfection of the 293FT packaging cells (Invitrogen) using Lipofectamine 2000 reagent and a Lentiviral packaging kit from Systems Biosciences (SBI), as previously described [4]. The viruses were collected by centrifuging 48-72 h post-transfection. Transduction of N27 cells was performed at a multiplicity of infection (MOI) of 1 in the presence of polybrene (6 μg/ml). Two days after transduction, GFP-expressing cells were sorted once per week for three weeks by flow cytometry, each time taking the brightest 1% of GFP-expressing cells to guarantee that 100% of the cells were GFP-positive.

The mouse dopaminergic MN9D cell line was kindly obtained from Dr. Syed Ali (National Center for Toxicological Research, Food and Drug Administration, Jefferson, AR) and cultured as described previously [5]. MN9D cells stably expressing human PK2 were created by stable transfection with the pCMV6-PK2-myc plasmid or empty vector control (Origene) using Lipofectamine 2000 reagent according to manufacturer's recommendations. The stable transfectants were selected in 400 μg/ml G418 and further maintained in 200 μg/ml G418 added to MN9D growth media. We used ciprofloxacin for mycoplasma elimination (GenHunter, Nashville, TN). The widely used N27 and MN9D neuronal cell models both represent a homogeneous population of TH-positive dopaminergic cells and are highly useful for studying degenerative mechanisms in PD [5, 6]. Primary neurons were obtained from the ventral mesencephalon of gestational 14-day-old (E14) mouse embryos as described previously [4]. Quantification of TH^+ cell counts and neuronal processes in primary neuronal cultures was performed as described previously [7]. TH^+ neurons and their processes were counted from three experiments in which over 8 fields were imaged in each well per group.

Caspase-3 activity, DNA fragmentation and ATP production

Enzymatic assays for caspase-3 activity were performed using acetyl-DEVD-amino-4-trifluoromethylcoumarin (Ac-DEVD-AFC, 25 μm) as the fluorometric substrate for the reaction,

as described previously [2, 8]. A Synergy-2 multi-mode microplate reader (BioTek Instruments, Inc) was used to detect fluorescent signals generated upon cleavage of the AFC peptide substrate by caspase-3 with excitation at 510 nm and emission at 400 nm. Caspase-3 activity was normalized by determining protein concentrations with the Bradford assay. For the DNA fragmentation assay, N27 cells were plated in 6-well plates at 0.8 x 10^6 cells/well and treated the next day. At the end of treatment, cells were gently lysed using the lysis buffer provided with the kit. Lysates were then spun down at 200 x g for 10 min to collect supernatants. The extent of DNA fragmentation was then quantified using the Cell Death Detection ELISA Plus assay kit (Roche Applied Science) according to previously published methods [2, 3, 8]. The plates were read by a Synergy-2 multi-mode microplate reader with absorbance at 405 nm and an ABTS solution (reference wavelength at ~490 nm) as a blank. The amount of protein in each sample lysate was used to normalize the raw absorbance values. For measurement of ATP production, cells were plated onto black-walled clear-bottom plates at 0.7 x 10^4 cells/well and exposed to 300 μM of MPP^+ for 16 h in 2% FBS medium. Following treatment, the plates were equilibrated for 15 min at room temperature. After equilibration, Cell Titer-Glo Luminescent reagent was added and then incubated at room temperature for 30 min to get a steady luminescence signal. Luminescence readout was recorded using the Synergy-2 multi-mode microplate reader.

Intracellular ROS generation

The fluorescent probe 2',7'-dichlorodi-hydrofluorescein diacetate (DCFH-DA) (Calbiochem) was used to determine the level of intracellular reactive oxygen species (iROS). N27 cells were treated with 200 μM MPP^+ in RPMI media with 2% FBS or co-treated with different doses of recombinant PK2 (rPK2), ranging from 5 to 50 nM, along with the MPP^+ treatment. After treatment for 8 h, the cells were washed with warm HBSS media, and then incubated with 100 μl of 40 μM DCFH-DA in HBSS for 1 h. The fluorescence intensity of the signal was determined using a Synergy-2 multi-mode microplate reader at an excitation of 485 nm and an emission of 530 nm. After subtracting the control fluorescent signal as background, an increase in fluorescence intensity of treatments was expressed as an increase in iROS, as previously described [9, 10].

Fluo-4 calcium mobilization assays

A Fluo-4 NW assay kit (Molecular probes) was used to test calcium mobilization in N27 cells and primary neurons resulting from nanomolar concentrations of rPK2. Cells were plated overnight into 96-well plates with their respective growth media. Cells were then washed with HBSS and incubated with the Fluo-4 NW dye. Each plate was then read kinetically using a Synergy-2 multi-mode microplate reader. After obtaining background readings by reading each

plate every 50 ms for 20 sec, rPK2 was injected at the indicated nanomolar concentrations. The net change in fluorescent signal (D_t), read every 50 ms for 3 min, was obtained after subtracting the background from the maximum signal for each sample.

Supplementary References

1. Clarkson ED*, et al.* Improvement of neurological deficits in 6-hydroxydopamine-lesioned rats after transplantation with allogeneic simian virus 40 large tumor antigen gene-induced immortalized dopamine cells. *Proceedings of the National Academy of Sciences of the United States of America* **95**, 1265-1270 (1998).

2. Kaul S, Kanthasamy A, Kitazawa M, Anantharam V, Kanthasamy AG. Caspase-3 dependent proteolytic activation of protein kinase C delta mediates and regulates 1-methyl-4-phenylpyridinium (MPP+)-induced apoptotic cell death in dopaminergic cells: relevance to oxidative stress in dopaminergic degeneration. *The European journal of neuroscience* **18**, 1387-1401 (2003).

3. Zhang D, Anantharam V, Kanthasamy A, Kanthasamy AG. Neuroprotective effect of protein kinase C delta inhibitor rottlerin in cell culture and animal models of Parkinson's disease. *The Journal of pharmacology and experimental therapeutics* **322**, 913-922 (2007).

4. Jin H, Kanthasamy A, Ghosh A, Yang Y, Anantharam V, Kanthasamy AG. alpha-Synuclein negatively regulates protein kinase Cdelta expression to suppress apoptosis in dopaminergic neurons by reducing p300 histone acetyltransferase activity. *The Journal of neuroscience : the official journal of the Society for Neuroscience* **31**, 2035-2051 (2011).

5. Jin H, Kanthasamy A, Anantharam V, Rana A, Kanthasamy AG. Transcriptional regulation of pro-apoptotic protein kinase Cdelta: implications for oxidative stress-induced neuronal cell death. *The Journal of biological chemistry* **286**, 19840-19859 (2011).

6. Zhang D, Kanthasamy A, Yang Y, Anantharam V, Kanthasamy A. Protein kinase C delta negatively regulates tyrosine hydroxylase activity and dopamine synthesis by enhancing protein phosphatase-2A activity in dopaminergic neurons. *The Journal of neuroscience : the official journal of the Society for Neuroscience* **27**, 5349-5362 (2007).

7. Ghosh A*, et al.* The peptidyl-prolyl isomerase Pin1 up-regulation and proapoptotic function in dopaminergic neurons: relevance to the pathogenesis of Parkinson disease. *The Journal of biological chemistry* **288**, 21955-21971 (2013).

8. Anantharam V, Kitazawa M, Wagner J, Kaul S, Kanthasamy AG. Caspase-3-dependent proteolytic cleavage of protein kinase Cdelta is essential for oxidative stress-mediated dopaminergic cell death after exposure to methylcyclopentadienyl manganese tricarbonyl. *The Journal of neuroscience : the official journal of the Society for Neuroscience* **22**, 1738-1751 (2002).

9. Qian L, *et al.* Microglia-mediated neurotoxicity is inhibited by morphine through an opioid receptor-independent reduction of NADPH oxidase activity. *J Immunol* **179**, 1198-1209 (2007).

10. Zhang W, *et al.* Aggregated alpha-synuclein activates microglia: a process leading to disease progression in Parkinson's disease. *Faseb J* **19**, 533-542 (2005).
